# A cost-based-plus pricing approach for repurposed tiratricol in the treatment of Allan-Herndon-Dudley syndrome

**DOI:** 10.1186/s13023-026-04269-7

**Published:** 2026-03-05

**Authors:** Evert Manders, Sarai Keestra, Wilbert Bannenberg, Vincent van der Wel, Saco de Visser, Carla Hollak

**Affiliations:** 1 Medicine for Society, Platform at Amsterdam University Medical Centre, Amsterdam, The Netherlands; 2 Department of Endocrinology and Metabolism, Amsterdam University Medical Centre, Amsterdam, The Netherlands; 3 RARE-NL, Dutch Collaboration of University Medical Centres Serving as a Hub for the National Centre for Future Affordable Sustainable Therapy Development, Amsterdam, The Netherlands; 4 Amsterdam Centre for Health Economics (ARCHE), A Collaboration Between the University of Amsterdam, Amsterdam UMC, UvA-EB (Faculty of Economics and Business, University of Amsterdam), and SEO Amsterdam Economics, Amsterdam, The Netherlands; 5 Paediatric Endocrinology, Emma Children’s Hospital, Amsterdam University Medical Centre, Amsterdam, The Netherlands; 6 Amsterdam Reproduction & Development Institute, Amsterdam University Medical Centre, Amsterdam, The Netherlands; 7 Pharmaceutical Accountability Foundation, Bergeijk, The Netherlands; 8 Orfenix BV, Leiden, The Netherlands; 9 Centre for Future Affordable & Sustainable Therapy Development (FAST), The Hague, The Netherlands

**Keywords:** Drug repurposing, Tiratricol, Allan-Herndon-Dudley syndrome, MCT8-defficiency, Drug pricing

## Abstract

**Background:**

Tiratricol, an old thyroid drug, has been repurposed as an orphan drug for Allan-Herndon-Dudley syndrome, a rare genetic disorder affecting thyroid hormone transport. Despite public contributions to its repurposing, the current marketing authorization holder, Egetis Therapeutics, has projected a price of €63.500–€95.000 per-patient-per year, significantly exceeding historical costs and straining already limited healthcare budgets. This raises concerns regarding affordability and the justification for charging high prices for medicines repurposed with public funding. This study evaluates a cost-based-plus pricing approach for repurposed tiratricol across different scenarios with varying cost structures and different assumptions about patient numbers. By transparently outlining key cost components, including R&D costs, cost-of-failure, and cost-of-capital, it proposes socially acceptable pricing that reflects genuine investment needs and a reasonable profit margin.

**Results:**

Under the maximum cost scenario, assuming fewer patients than projected by the company itself, a price of €27.600 per-patient-per-year is derived. Minimum cost scenarios and those projecting a higher number of patients yield prices ranging from €5.300 to €16.500.

**Conclusions:**

Given the substantial public role in tiratricol’s repurposing, this study argues in favour of the lower end of the price range, while acknowledging the need for sustainable access through formal registration, which justifies a price higher than that of the original product. This research highlights the broader ethical and economic implications of orphan drug pricing, advocating for evidence-based policies that promote transparency in cost structures. This can help ensure a more predictable, sustainable, and socially responsible business model for repurposed medicines.

**Supplementary information:**

The online version contains supplementary material available at 10.1186/s13023-026-04269-7.

## Introduction

Tiratricol, or triiodothyroacetic acid, also known as TRIAC, is an acetic acid analogue of the endogenous thyroid hormone triiodothyronine (T3) and was first synthesised in 1953 [1]. By 1974, it was approved for managing thyroid hormone resistance syndrome (RTH) and was marketed exclusively in France under the brand name Téatrois® [2, 3]. Initially produced by Théranol-Deglaude, manufacturing later shifted to Cenexi, with DB Pharma managing distribution. In other territories, tiratricol, under different brand names, has been available for decades as an over-the-counter dietary supplement marketed for weight loss [4–7].

In 2014, tiratricol was reported as a potential therapy for Allan-Herndon-Dudley syndrome (AHDS), a rare and severe X-linked disorder caused by mutations in the SLC16A2 gene encoding the monocarboxylate transporter 8 (MCT8) transporter protein (Additional file 1), following research conducted by Dutch researchers at the Erasmus Medical Centre (Erasmus MC) [8, 9]. These mutations impair the transport of thyroid hormones (THs) into various tissues, including the brain [10, 11], thereby disrupting neurodevelopment [12]. A deficiency of T3 in the brain leads to severe intellectual disabilities, while dysregulated thyroid hormone homeostasis, due to impaired feedback regulation, results in excess T3 in peripheral tissues, causing symptoms such as muscle wasting, tachycardia, and weight loss, collectively known as thyrotoxicosis [10, 11, 13–15]. Unlike T3, tiratricol bypasses the defective MCT8 transporter in AHDS patients and directly enters neuronal cells, alleviating critical thyroid hormone deficiencies in the brain, making it a promising candidate for AHDS treatment [16–22]. Encouraged by the promising results of in vitro and in vivo studies [8, 23], researchers at Erasmus MC in Rotterdam, Netherlands, initiated a multinational Phase IIb open-label, single-arm trial in 2014. This trial, known as the TRIAC I trial (NCT02060474), enrolled 46 patients from nine countries and was funded by ZonMW (the Netherlands Organisation for Health Research and Development), along with other public and charitable organisations [24]. The results, published in The Lancet Diabetes & Endocrinology, highlighted tiratricol’s potential for effectively treating AHDS [20].

Recognising tiratricol’s potential, Medical Need Europe AB acquired its rights in 2017 and partnered with Erasmus MC to advance its development [25, 26]. That same year, tiratricol received orphan drug designation from the European Medicines Agency (EMA) [27], followed by the U.S. Food and Drug Administration (FDA) in January 2019 [28]. After several sponsorship transfers, the drug was rebranded as Emcitate®, and the Swedish pharmaceutical company Egetis Therapeutics became the marketing authorisation holder, which subsequently withdrew the drug from the market [2, 3, 29–32]. While these acquisitions and rebranding were taking place, subsequent trials expanded upon the results of the 2014 TRIAC I trial. The TRIAC II trial (NCT02396459), launched in 2020, focuses on investigating neurodevelopmental outcomes in young children aged ≤30 months with AHDS [33]. The trial also monitors safety and serum T3 levels as secondary outcomes. Similarly, the ReTRIACt Study (NCT05579), initiated in 2023, is a Phase III double-blind, placebo-controlled trial evaluating the effects of discontinuing tiratricol in AHDS patients [34]. Prior to the publication of the results of these studies, Emcitate® received a positive opinion from the Committee for Medicinal Products for Human Use (CHMP) in December 2024 [35], marking a key milestone in its regulatory approval process and paving the way for full marketing authorisation in the European Union (EU), officially granted in February 2025 [36].

While these clinical and regulatory advancements represent a significant breakthrough for AHDS patients, they also bring attention to a contentious issue: the pricing of tiratricol. After its withdrawal from the French market, concerns about pricing for AHDS patients quickly arose [37]. Unlike Téatrois®, which was registered for thyroid disorders, Emcitate® has received orphan drug designation for treating a severe and rare disease with limited treatment options. This, along with the monopoly position created by the withdrawal of Téatrois® from the market, allows Egetis to set significantly higher prices. Under Théranol-Deglaude, tiratricol was priced at €35 per 100 tablets. After Cenexi and DB Pharma took over, the price increased to €65 per 100 tablets, likely due to rising manufacturing and distribution costs [2, 3]. However, in its 2021 annual report, Egetis projected that, given “an addressable population of 10.000–15.000 potential patients, the total theoretical market potential for the product will be over USD 1 billion, even with cautious assumptions about market penetration” [38]. This estimate translates to a price of $66.667–$100.000 or ~€63.500–€95.000 per-patient-per-year (PPPY) (€2.670–€3.995 per 100 tablets, assuming 60 kg body weight, 38 µg/kg/day). Furthermore, Egetis suggests even higher pricing, referring to orphan drugs priced between $375.000–$750.000 PPPY in the U.S. and €250.000–€600.000 PPPY in Europe. Consequently, while the development and market launch of tiratricol for AHDS represent a critical advancement for patients suffering from the disease, the stark contrast in pricing raises serious questions about drug accessibility, the role of public investment in price, and the extent of profit margins in rare disease therapies. Moreover, the case also highlights limitations of conventional health technology assessments (HTAs), cost-effectiveness analyses, and willingness-to-pay (WTP) thresholds when appraising medicines that were previously inexpensive but are repurposed with public support and reintroduced at high prices.

This study aims to evaluate the potential of a cost-based-plus pricing model for repurposed tiratricol and, in doing so, contribute to the development of socially responsible pricing strategies for rare disease therapies that balance affordability with sufficient incentives for sustainable drug repurposing. By analysing multiple scenarios within the EU context, reflecting variation in underlying cost structures, this research seeks to establish a transparent, evidence-based framework that directly links pricing to actual investments, public contributions, and sustainable healthcare financing, while also providing a cost-based rationale to complement value-based approaches. This can support clinical and economic evaluations in HTAs, particularly for repurposed medicines that were already inexpensive before being relaunched.

## Methods

To evaluate the feasibility of a cost-based-plus pricing model for repurposed tiratricol, a case analysis was conducted to examine the drug’s repurposing trajectory, providing essential context for the scenario analysis. This analysis focused on funding sources, the development pathway, and key financial and regulatory milestones that have shaped the commercialisation of tiratricol.

Based on these findings, multiple pricing scenarios were developed to assess the impact of different funding contributions on the final drug price within the proposed pricing framework. These scenarios incorporate variations in research and development (R&D) cost estimates and the eligible patient population.

### Case analysis

To construct an accurate scenario analysis, the historical development and funding sources of tiratricol for AHDS were examined using a combination of academic and non-academic sources, enabling an evaluation of public and private contributions to its research, clinical development, and commercialisation.

A key source of historical information was the French Thyroid Forum (Forum Thyroïde), an online patient and expert community documenting discussions on tiratricol’s historical use and availability in France. This forum provided valuable insights into early access to tiratricol, its pricing history, and its transition from a thyroid treatment to an orphan drug candidate for AHDS [2, 3]. Statements from this forum served as a starting point rather than definitive evidence and were cross-referenced with official sources, including regulatory filings and corporate reports, to ensure accuracy and contextual validity.

Corporate data were obtained from annual reports and financial disclosures from Egetis Therapeutics and its predecessor companies. These reports provided insights into acquisitions and corporate decisions relevant to tiratricol’s repurposing. Similarly, orphan drug designations granted by the EMA and FDA were reviewed to assess the regulatory incentives provided to tiratricol as part of its repositioning for AHDS.

A non-systematic search on Google Scholar was conducted to identify non-clinical and clinical studies on tiratricol for AHDS. This search was supplemented by a systematic search on ClinicalTrials.gov to retrieve clinical trials evaluating tiratricol’s efficacy. In addition, the publicly available European Public Assessment Report (EPAR) for Emcitate was reviewed to identify studies used for registration of Emcitate in the EU [39]. The “Funding,” “Acknowledgements,” and “Conflicts of Interest” sections of the identified studies were examined to trace funding sources, particularly those from governmental and non-profit organisations. Additionally, funding records from the ZonMW and Eurostar grant databases were reviewed to identify financial support from public institutions for tiratricol-related research.

### Drug pricing framework

**Fig. 1:**
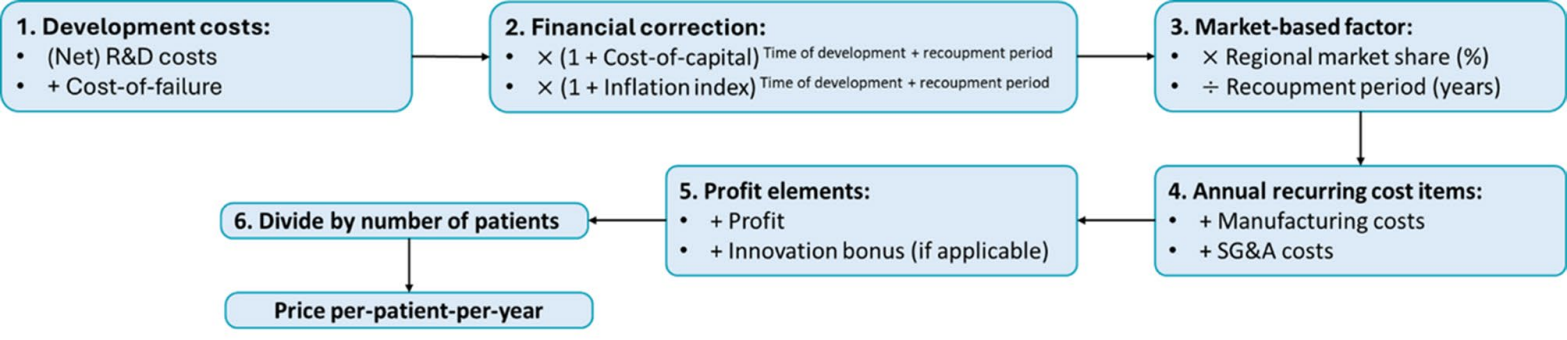
Visual overview of the pricing approach

The pricing structure for repurposed tiratricol is analysed through a cost-based-plus framework, illustrated in Fig. 1, detailed in Table 1 and defined by the equations below. As the pricing framework calculates a price PPPY, all components are expressed as the annual amounts to be recovered for a defined target region, in this study the EU context, over a specified recoupment period.

**Table 1:** Overview of the frameworks’ components

Category:	Components:	Cost type:	Parameter:	Definition:
1. Development costs	(Net) R&D costs	Fixed	Lead development costs	Costs associated with early discovery and translational activities required to nominate a viable drug candidate for further development, including target identification and validation, hit discovery, lead optimisation, and candidate selection
			Non-clinical development costs	Costs associated with early-stage research activities. Includes discovery, in vitro and in vivo studies, pharmacology, and toxicology assessments.
			In-licensing costs	Costs incurred when acquiring the rights to develop, manufacture, or commercialize a drug candidate from an external entity.
			R&D operational costs	Indirect but essential expenses enabling the functioning of R&D activities, including staff salaries, laboratory facilities, utilities, general consumables, project management, and IT systems. These costs support multiple development activities and may be partially attributable to specific programmes or trials through internal allocation methods.
			Legal development costs	Legal expenses incurred during the development phase to secure product readiness and manage development- and launch-related risks, including freedom-to-operate analyses, IP strategy and filings, compliance reviews, and contractual negotiations.
			Product development costs	Fixed costs required to convert a drug candidate into a trial-ready and manufacturable product, including GMP process development, formulation and delivery optimisation, analytical method development and validation, production of engineering and clinical batches, process validation, equipment qualification, and preparation of regulatory quality documentation (e.g., ASMF, IMPD, BHR, or equivalent dossiers).
			Clinical development costs	Costs related to conducting clinical trials (Phases I–III), including trial design, site management, investigator fees, patient recruitment, data monitoring, and clinical operations.
			Early and expanded access costs	Costs associated with providing investigational drugs to patients outside of clinical trials before marketing authorization, under expanded access, compassionate use, or early access programs.
			Regulatory development costs	Expenses related to regulatory strategy, scientific advice, interactions with regulatory authorities, and preparation and submission of marketing authorisation dossiers, including external consultancy services.
		Fixed deduction (cost offset)*	Public investments & charitable funding	External funding from governments, public institutions, or nonprofit organizations that reduces the net private cost of R&D. As these contributions do not require repayment or return, they may be deducted when calculating recoverable investment.
	Cost-of-failure	Fixed	Cost-of-failure correction factor**	A factor representing the ratio of cost-of-failure to R&D costs, used to account for expenses incurred during the R&D process for drugs that ultimately do not reach the market due to failure at any stage. By distributing these costs across successful products, this factor reflects the inherent financial risk of pharmaceutical innovation.
2. Financial correction factors	Cost-of-capital	Fixed	WACC (%)	A metric representing the average cost a company incurs to finance its operations through a combination of equity and debt. Expressed as a percentage of invested capital, it reflects the blended cost of raising funds from equity investors and creditors. Often used as a discount factor in financial evaluations.
			Cost-of-capital (development phase)	The capital charge applied to out-of-pocket R&D expenditures and cost-of-failure during the development period (from research initiation until market authorization). It reflects the opportunity cost of tying up capital before any revenue is generated.
			Cost-of-capital (recoupment phase)	The capital charge applied during the recoupment period on the outstanding unrecovered balance of R&D investment. It reflects the ongoing financing cost of carrying unrecovered capital until full cost recovery is achieved through sales.
	Inflation index	N/A	GDP deflator (%)	A measure used to adjust nominal GDP to reflect inflation rates, thereby accounting for inflation when setting or evaluating costs.
	Time of development	N/A	Development period (years)	The duration from the initial research phase to obtaining market authorization for a new drug.
3. Market-based factor	Market allocation factor	N/A	Regional market share (%)	The proportion of the revenue base attributed to the target region, used to allocate global development costs. This reflects the region’s relative economic weight and revenue potential.
			Recoupment period (years)	The timeframe within which the company aims to recover its initial investment costs through sales revenue.
4. Annual recurring cost items	Manufacturing costs	Recurring	Manufacturing cost per treatment	The cost associated with manufacturing each individual unit of the product, including raw materials, labour, and overhead expenses directly tied to production.
			Manufacturing volume	The quantity of the product that needs to be produced annually to meet market demand. Influenced by the number of treatments required per-patient-per-year and the number of patients.
	SG&A costs	Recurring	Cost-of-sales	Sales-related expenses, such as distribution, marketing, and sales force efforts.
			Operational costs	Indirect costs associated with ongoing company operations, such as corporate administration, HR, finance, legal, compliance, and IT services.
			Overhead costs	Costs such as rent, utilities, and depreciation.
			Post-authorization obligations	Costs incurred after market approval, including participation in patient registries, long-term safety and effectiveness monitoring, and the collection of real-world evidence as required by regulators or payers.
			Regulatory maintenance	Ongoing expenses related to maintaining product authorization, submitting periodic safety updates (PSURs), variations, renewals, and compliance with evolving regulatory requirements.
			Patent maintenance costs	Recurring legal expenses required to preserve IP rights after the initial patent strategy is implemented. Includes renewals, jurisdictional filings, and defence activities such as oppositions.
			Licensing obligations	Incremental legal or financial obligations under licensing agreements, such as royalty payments per unit sold or milestone fees tied to specific development or commercialization events.
5. Profit elements	Profit	Recurring	Profit margin (%)	Bonus added to ensure a baseline level of profitability, supporting financial stability and continued investment.
	Innovation bonus	Recurring	Value-driven premium (%)***	An additional pricing bonus, granted based on the drug’s clinical effectiveness and innovation, reflecting its added therapeutic value and novelty.
6. Patient population	Number of patients	N/A	Number of patients receiving treatment***	The total number of patients expected to receive the drug treatment. This figure is shaped by factors such as disease incidence, prevalence, mortality rates, competition, and market penetration.

*While technically income, development cost offsets (e.g., public and charitable contributions) are accounted for as fixed deductions in the pricing model

**Alternatively, cost-of-failure may be represented as an absolute monetary value

***Dynamically adjustable based on emerging (clinical) data


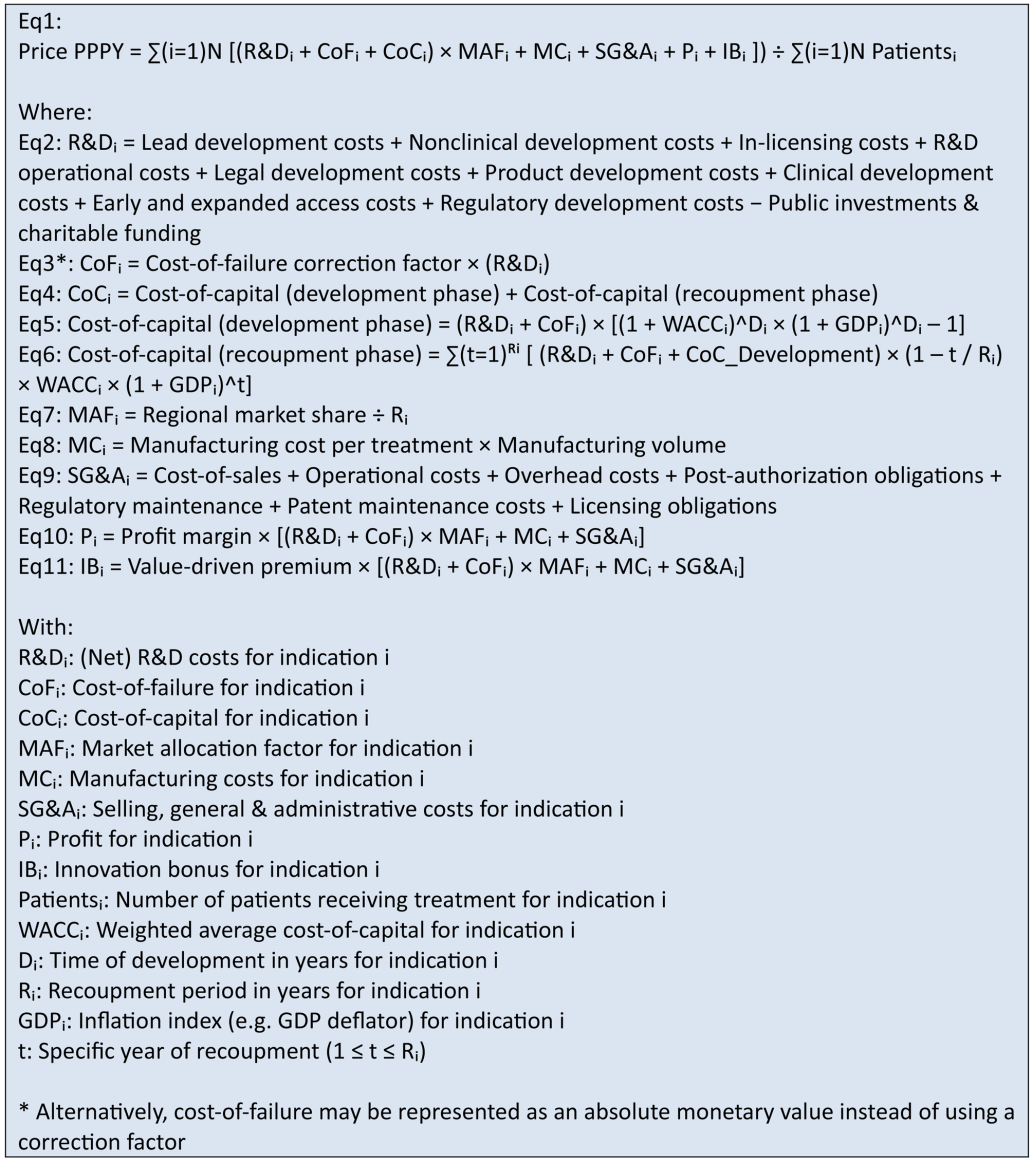


The framework builds on established drug pricing models proposed by the International Association of Mutual Benefit Societies (AIM, 2021), Uyl-de Groot and Löwenberg (2018), Nuijten and Vis (2016), and van der Schans et al. (2022) [40–43]. These cost-based pricing approaches currently function as proposed analytical frameworks rather than as formal institutional pricing or reimbursement instruments. These models were analysed in detail, and four of them were previously applied in the case of lumasiran to illustrate their practical use [44, 45].

First, fixed costs of developing a new molecular entity (NME) or repurposing an existing drug are incorporated. These include R&D costs together with presumed costs of unsuccessful projects (cost-of-failure). Although R&D costs are built up from multiple parameters (Table 1), in the absence of detailed data a lump-sum estimate is assigned for the further development of tiratricol for AHDS. Development cost offsets, such as public investments and charitable funding, are deducted to reflect the role of external contributions in reducing the overall R&D investment that needs to be recovered through pricing.

The resulting net development costs are subsequently corrected for the cost-of-capital and inflation, accruing over both the development and recoupment periods. Following the AIM model, these development costs (R&D, cost-of-failure, and cost-of-capital corrected for inflation) are further scaled according to the target region’s market share and the expected recoupment period. This ensures that pricing reflects both the size of the region’s market and the timeframe over which investments are to be recovered. For this initial application, the population share of the EU relative to the total population of OECD (Organisation for Economic Co-operation and Development) countries (≈1,38 billion in 2023 [46]) is used, under the assumption that OECD countries represent the primary market for innovative pharmaceuticals due to their high healthcare spending and uptake of new medicines [47].

Next, annual recurring cost items are added, including manufacturing costs and selling, general, and administrative (SG&A) costs. As with R&D, SG&A is normally composed of multiple parameters (Table 1); however, due to limited transparency a lump-sum estimate is assigned.

A profit margin is then included to incorporate financial incentives for pharmaceutical companies to continue drug development. In this framework, this margin is applied to net R&D costs, cost-of-failure, manufacturing costs, and SG&A costs. Development costs are not further capitalized or adjusted for inflation, but are adjusted for the regional market share and recoupment period

Finally, the framework allows for a value-driven innovation bonus, to further stimulate and reward therapeutic breakthroughs that deliver substantial clinical benefits compared to existing treatments. This bonus is applied in the same manner as the profit margin: it is calculated on non-capitalized net R&D costs and cost-of-failure, corrected for regional market share and recoupment period, and added on top of manufacturing and SG&A costs.

For this analysis, a sensitivity analysis was conducted using RStudio to evaluate the impact of various parameters on the price PPPY. Each parameter was varied by ±20%, and the resulting changes in outcomes were analysed to identify the most influential cost drivers.

### Scenario-analysis

Distinct pricing scenarios for repurposed tiratricol in EU context are analysed using case-specific assumptions and reference values for operationalisation. The primary scenarios explore both maximum and minimum cost structures and incorporate different assumptions regarding the number of patients receiving treatment, while accounting for the role and financing of public entities in the repurposing of tiratricol for AHDS. Variations are introduced in the assigned cost-of-failure and the number of patients receiving treatment. In all primary scenarios, a recoupment period of ten years is assumed, consistent with evidence that roughly half of new drugs reach breakeven within this timeframe [48], with the exclusion of a value-driven premium, recognising that the benefits associated with repurposed tiratricol have been supported by publicly funded research.

In addition to these primary scenarios, secondary analyses introduce variations in the assigned profit margin to assess their impact on pricing outcomes (Additional file 2). Furthermore, secondary analyses consider the scenario in which the company would independently finance the entire development process without any public or charitable contributions (Additional file 2). This scenario assumes high R&D costs, significant cost-of-failure expenses, and a short recoupment period of three years, during which the company is expected to recover its development costs. This exercise, intended to explore the cost structure of a fully privately funded development process, also incorporates variations in the eligible patient population. In all scenarios, manufacturing costs are derived from historical tiratricol tablet prices, based on which an allowance for SG&A costs is assigned.

## Results

### Case analysis

The development of tiratricol for AHDS has been shaped by a combination of public investments, charitable funding, private investments and corporate acquisitions. Table 2 outlines the key events in tiratricol’s trajectory, from its initial discovery into its most recent regulatory advancements. These milestones highlight the transition from a treatment for thyroid disorders to an orphan drug candidate for AHDS, facilitated by scientific discoveries, funding support, and regulatory incentives.

**Table 2:** Timeline of tiratricol’s development for AHDS

Year:	Event:	References:
1953	Tiratricol is first synthesised and reported to be highly biologically active in a similar manner to endogenous T3	[1]
1950’s-60’s	Following the discovery of Tiratricol, its effectiveness is investigated as a therapy for hypothyroidism.	[18, 19, 49–52]
1974	Tiratricol is approved for managing thyroid hormone resistance syndrome (RTH) and marketed in France as Téatrois®.	[2, 3]
2014	Early in vitro and in vivo studies conducted at the university hospital Erasmus MC demonstrate Tiratricol’s potential for treating Allan-Herndon-Dudley Syndrome (AHDS); researchers from the Erasmus Medical Centre (Erasmus MC) initiate a multinational clinical trial with 46 patients from nine countries to investigate Tiratricol’s efficacy for AHDS.	[8, 24]
2017	Medical Need Europe AB acquires rights to Tiratricol and partners with Erasmus MC to advance development; Tiratricol receives orphan drug designation from the European Medicines Agency (EMA).	[25–27]
2018	Sponsorship of Tiratricol is transferred to MN Development AB, later rebranded as Rare Thyroid Therapeutics International AB (RTTI); Téatrois® is rebranded as Emcitate®.	[3, 25]
2019	The price of tiratricol rises in France, increasing from €65 to over €184 per 100 tablets; The U.S. Food and Drug Administration (FDA) grants orphan drug designation for Tiratricol; Results from the TRIAC I trial are published in *The Lancet Diabetes & Endocrinology*, highlighting its efficacy and safety for AHDS.	[3, 20, 28]
2020	RTTI is acquired by PledPharma AB, which subsequently rebrands itself as Egetis Therapeutics AB; Téatrois® is withdrawn from the French market; The TRIAC II trial, an open-label Phase II trial, is launched to investigate neurodevelopmental outcomes in children aged ≤30 months with AHDS; Egetis acquires Rare Paediatric Disease Designation (RPDD) in the USA.	[29–33, 53]
2023	The ReTRIACt Study, a double-blind, placebo-controlled Phase III trial, is initiated to assess the impact of withdrawing Tiratricol in patients with AHDS.	[34]
2024	Emcitate® receives a positive opinion from the Committee for Medicinal Products for Human Use (CHMP), paving the way for EMA market authorization.	[35, 53]

Medical Need Europe AB initially obtained orphan drug designation for tiratricol. However, in 2018, sponsorship was transferred to its subsidiary, MN Development AB, which later rebranded as Rare Thyroid Therapeutics International AB (RTTI) [27]. Under RTTI’s ownership, the drug was renamed Emcitate® [2, 3]. As RTTI advanced the development of Emcitate®, the price increased further, exceeding €184 for 100 tablets. This rise reflected the company’s investments in regulatory approvals for AHDS and production modernisation [3]. In November 2020, RTTI was acquired by PledPharma AB, which subsequently integrated Emcitate® into its portfolio [29]. Following the acquisition, PledPharma rebranded as Egetis Therapeutics AB [30] and simultaneously withdrew tiratricol from the French market [3, 32].

A wide range of public, private, and charitable funders have provided support for non-clinical research, clinical trials, and long-term efficacy studies of tiratricol for AHDS (Table 3) (Additional file 3). Notably, the early-stage non-clinical research that established tiratricol’s potential in MCT8 deficiency were not conducted by Egetis and funded by charitable foundations and multinational research organisations [8, 18, 19]. These findings enabled subsequent clinical trial investigations, which also received public and charitable funding. The TRIAC I trial, which provided critical clinical evidence for tiratricol’s efficacy in AHDS patients [20], and the retrospective cohort studies by Groeneweg et al. (2020) and van Geest et al. (2022), were also supported by public and charitable funding sources [21, 22]. By contrast, Egetis itself conducted only a limited number of small-scale (non-)clinical studies, as documented in the EPAR [39], as well as the ongoing TRIAC Trial II (NCT02396459) (Where Erasmus MC is also a collaborator) and the ReTRIACt Study (NCT05579). These studies, together with the fact that tiratricol had previously received national authorization in France, provided the evidentiary basis for a hybrid marketing authorization application under Article 10(3) of Directive 2001/83/EC [39].

**Table 3:** Public and charitable funding for tiratricol repurposing studies

Organization:	Support:	Details:	Study:
Sherman Foundation	Research, clinical trial and study funding	Supported (non-clinical) studies on tiratricol’s mechanism and therapeutic potential; co-funded TRIAC Phase II trial and retrospective cohort studies on MCT8 deficiency.	[8, 15, 18–22]
ZonMw (Netherlands Organisation for Health Research and Development)	Research, clinical trial and study funding	Supported studies on tiratricol’s mechanism and therapeutic potential; co-funded TRIAC Phase II trial and retrospective cohort study on MCT8 deficiency.	[18–21]
Smile Foundation	Research funding	Supported non-clinical study on tiratricol’s mechanism and therapeutic potential.	[8]
Jérôme Lejeune Foundation	Research funding	Supported non-clinical study on tiratricol’s mechanism and therapeutic potential.	[8]
BMBF (E-RARE Project Thyronerve)	Research funding	Supported non-clinical study on tiratricol’s mechanism and therapeutic potential.	[8]
NeMO Foundation	Clinical trial funding	Co-funded TRIAC Phase II trial, assessing tiratricol’s safety and efficacy.	[20]
Toulouse University Hospital	Clinical trial funding	Contributed funding and institutional support to the TRIAC Phase II multinational trial.	[20]
Una Vita Rara ONLUS	Charitable funding	Co-funded TRIAC Phase II trial, assessing tiratricol’s safety and efficacy.	[20]
Wellcome Trust	Clinical trial funding	Co-funded TRIAC Phase II trial, assessing tiratricol’s safety and efficacy; Facilitated international collaboration	[20, 22]
NIHR Cambridge Biomedical Centre	Clinical trial funding	Co-funded TRIAC Phase II trial, assessing tiratricol’s safety and efficacy; Facilitated patient recruitment and study activities.	[20, 22]
Eurostars	Study funding	Supported studies on tiratricol’s mechanism and therapeutic potential; co-funded study on the long-term efficacy of tiratricol in children and adults with MCT8 deficiency.	[15, 22]

This external funding likely reduced the financial burden on private-sector investors and mitigated development risks. Beyond direct financial contributions, institutional support played a role in facilitating patient recruitment, streamlining trial operations, and enabling long-term follow-up studies. For instance, the NIHR Cambridge Biomedical Centre and Toulouse University Hospital provided infrastructure and logistical assistance for multinational trials, ensuring efficient patient enrolment and high-quality data collection.

During and after the conduct of these studies, and following tiratricol’s withdrawal from the market, Egetis made the drug available to AHDS patients through managed access programmes, enabling over 230 patients worldwide to receive tiratricol on a compassionate-use basis while regulatory approvals were being pursued [53].

Regulatory incentives further influenced the repurposing of tiratricol. In 2020, Egetis received a Rare Paediatric Disease Designation (RPDD) for tiratricol in the United States, granting the company the right to a Priority Review Voucher (PRV) upon U.S. approval [38, 53]. PRVs enable expedited FDA review of a future drug application and can be sold to other pharmaceutical companies for substantial amounts (Table 4), representing a potentially significant cost offset that could impact the financial modelling of repurposed tiratricol [54–58]. In its 2021 annual report Egetis states it expects this PRV to be worth $50 million [38].

**Table 4:** Recent priority review voucher (PRV) sales

Price (USD):	Involved party:	Reference:
$21,1 million	Pharming	[54]
$102 million	Sarepta Therapeutics	[55]
$103 million	Valneva	[56]
$150 million	PTC Therapeutics	[57]
$158 million	Ipsen	[58]

Egetis’ annual report for 2024 specifies that the company is required to pay the former owners of RTTI and Erasmus MC 3% and 10% of net sales of the product, respectively [53]. Additionally, the former owners are entitled to a one-time payment equal to 50% of the net proceeds in the event of a future sale of the U.S. PRV. The “Conflict of Interests” statements from the identified studies highlight that none of these payments go to the individual researchers involved in the product’s development.

Egetis emphasized in its 2021 annual report that it focuses on countries with healthcare standards comparable to those of the Western world [38]. This approach supports adjusting the EU’s regional market share using OECD countries, as applied in this analysis. Furthermore, Egetis estimates an addressable population of 10.000–15.000 potential patients, based on the prevalence of AHDS reported by Groeneweg et al. (2020) [21]. The company also highlights its intention to follow value-based pricing principles in Europe, which are based on costs in relation to Quality Adjusted Life Years (QALYs). Following these principles, it is estimated that “*the total theoretical market potential for the product will be over USD 1 billion even with cautious assumptions about market penetration*”. Additionally, the report states that *“it would be possible, once market approval has been obtained, to distribute information about Emcitate to relevant doctors with very limited sales and marketing efforts required”* [38].

Finally, the case analysis highlights that Egetis obtained Orphan Drug Designation for tiratricol for the treatment of Resistance to Thyroid Hormone Beta (RTH-β) from both the FDA and the EMA in 2022. The company plans to pursue further development and regulatory approval of Emcitate specifically for RTH-β treatment, which could eventually expand the eligible patient population for tiratricol [38, 53].

### Pricing scenario’s:

Based on the results of the case analysis, various scenarios have been developed to calculate a cost-based price for repurposed tiratricol for AHDS. Table 5 presents the four scenarios, which consider the role of public funding and vary based on the assigned cost-of-failure, and the eligible patient population. The other reference scenarios, which either assume a fully privately funded repurposing trajectory or a higher profit margin, are presented in Additional file 2.

**Table 5:** Overview of the primary scenarios

Items:	Scenario 1a:	Scenario 1b:	Scenario 1c:	Scenario 1d:
**1. Development costs**				
(Net) research & development costs	€50 million	€50 million	€50 million	€50 million
Cost-of-failure	None, all risk is presumed to be borne by public entities and charitable foundations	0,84 times the assigned (net) R&D costs	None, all risk is presumed to be borne by public entities and charitable foundations	0,84 times the assigned (net) R&D costs
**2. Financial correction factors**				
Cost-of-capital	Calculated at a discount rate of 10,5%	Calculated at a discount rate of 10,5%	Calculated at a discount rate of 10,5%	Calculated at a discount rate of 10,5%
Development period (years)	10	10	10	10
**3. Market-based factor**				
Regional market share (%)	32,61%	32,61%	32,61%	32,61%
Recoupment period (years)	10	10	10	10
**4. Annual recurring cost items**				
Manufacturing costs	Benchmarked at €0,65 per tablet and 2.378 tablets per-patient-per-year	Benchmarked at €0,65 per tablet and 2.378 tablets per-patient-per-year	Benchmarked at €0,65 per tablet and 2.378 tablets per-patient-per-year	Benchmarked at €0,65 per tablet and 2.378 tablets per-patient-per-year
SG&A costs	Benchmarked at 90% of manufacturing costs	Benchmarked at 90% of manufacturing costs	Benchmarked at 90% of manufacturing costs	Benchmarked at 90% of manufacturing costs
**5. Profit elements**				
Profit (%)*	8%	8%	8%	8%
Innovation bonus	None, as the value repurposed tiratricol provides to patients primarily stems from public research	None, as the value repurposed tiratricol provides to patients primarily stems from public research	None, as the value repurposed tiratricol provides to patients primarily stems from public research	None, as the value repurposed tiratricol provides to patients primarily stems from public research
**6. Patient population**				
Number of patients	500	500	3.129	3.129

*Applied to non-capitalized development costs (net R&D costs and cost-of-failure), not adjusted for inflation, but corrected for the regional market share and the assigned recoupment period. This bonus is further granted on top of the out-of-pocket annual recurring cost items. Reflects the annual amount that is provides as bonus each year

**(Net) R&D costs:** Published estimates for bringing a NME to market vary due to methodological differences. Some studies include cost-of-capital and cost-of-failure, accounted for separately in this framework, while others exclude them [59–66]. R&D costs also depend on the product type and therapeutic area. Orphan drug development is generally considered less expensive due to smaller patient populations and fewer, smaller trials, which lower overall costs [60, 67–69].

Jayasundara et al. (2019) estimated clinical development costs for an orphan drug at $166 million, covering direct clinical trial (Phase 1–3) costs but excluding non-clinical expenses such as laboratory research, animal studies, and manufacturing setup [68]. Regulatory costs and expenses related to compassionate use programs, which Egetis has implemented, are also not included. According to DiMasi et al. (2016), non-clinical costs account for 44,6% of total development costs [59], meaning nearly half of total expenditure occurs before clinical trials—costs not captured in Jayasundara et al.‘s (2019) estimate.

In contrast, Gupta Strategists (2019) provide a broader estimate, placing total orphan drug development costs at $600 million, which includes $126 million in out-of-pocket R&D costs (21%), $210 million for cost-of-failure (35%), and $264 million (44%) for cost-of-capital [60]. Given these disparities in orphan drug R&D cost estimates, a cost item of €135 million can be assumed for a fully privately financed development trajectory (Additional file 2), a figure that lies between the estimates provided by Jayasundara et al. (2019) and Gupta Strategists (2019). Although the initial estimate focuses only on clinical development costs, it can be argued that the monetary value of the obtained PRV (Table 4) offsets any possible underestimation.

These R&D costs are adjusted downward to account for public investments and contributions in tiratricol’s repurposing. Case analyses show that both non-clinical and clinical research received public funding, with the majority of non-clinical studies conducted by public institutions, while Egetis itself only carried out a limited number of small-scale (non-)clinical studies (Table 3, Additional file 3). Quantifying these contributions is challenging, particularly when considering indirect costs, such as full-time equivalents (FTEs) of public institution staff, which might otherwise be classified as R&D expenses for Egetis. However, this monetary and institutional support underscores that Egetis did not bear the full cost of the R&D process.

Since non-clinical costs are estimated to make up 44.6% of total R&D expenses [59], and the bulk of these costs were covered by public contributions rather than by Egetis, applying this proportion to the €135 million maximum scenario estimate reduces total R&D costs to ~€75 million. This estimate may still be high, given additional public funding during clinical development. Consequently, net R&D costs are estimated to be closer to €50 million.

**Cost-of-failure:** The cost-of-failure for orphan drugs is estimated to be 1,67 times the out-of-pocket R&D costs, as reported by Gupta Strategists [60]. This estimation is applied in the scenario which assumes no contributions of public or charitable funding in tiratricols development (Additional file 2). Scenarios 1a and 1c exclude the cost-of-failure entirely, reflecting the role of public entities in bearing a substantial portion of the development risk. However, in Scenarios 1b and 1d, a low cost-of-failure allocation (0,84 times the assigned out-of-pocket R&D costs) is included. This adjustment is based on the argument that certain aspects of the development process still involved financial risk and uncertainty for the pharmaceutical company, particularly in later stages where public involvement was less prominent.

**Cost-of-capital:** Following DiMasi et al. (2016) and Wouters et al. (2020), a real weighted average cost-of-capital (WACC) of 10,5% is applied to account for the opportunity cost of funds tied up during both the development (pre-market) and recoupment (post-launch) periods [59, 62]. Since a real discount rate is used, inflation is not separately included in the calculations.

**Manufacturing costs:** Manufacturing costs are benchmarked at €0,65 per tablet, based on tiratricol’s reported cost under Cenexi. This price was chosen over the lower Theranol-Deglaude price (€0,35 per tablet) and the higher RTTI price (€1,84 per tablet) for two key reasons. First, the production line was modernized under Cenexi and RTTI—before Egetis acquired the company—making the lower Theranol-Deglaude price less relevant. Second, the higher RTTI price reflects investments in regulatory approvals and clinical trials for AHDS, which are separately accounted for in this analysis. While inflation has risen since DB Pharma managed distribution, the €0,65 price likely included a profit margin for both the manufacturer and distributor, potentially offsetting inflation’s impact.

For this analysis, average patient use is estimated at 6,5 tablets per day, based on a required dosage of 38 μg/kg of body weight [22] and an average body weight of 60 kg, totalling 2.378 tablets per year.

**SG&A costs:** Empirical benchmarks show that SG&A expenses account for a similar share of revenue as cost-of-goods-sold (COGS). Ledley et al. report median SG&A equal to 28,2% of revenue and COGS equal to 23,5% of revenue, while Chandra et al. (2024) similarly find SG&A averaging ~24% of revenue and COGS ~23% of revenue [70, 71]. Chandra et al. further estimates that about 40% of SG&A relates to sales and marketing, and ~60% to other general functions [71].

Building on this evidence, SG&A is modelled relative to manufacturing costs. To address concerns about excessive marketing in the pharmaceutical sector, and consistent with Uyl-de Groot and Löwenberg (2018), sales-related expenses are capped at 30% of manufacturing costs [41]. Non-sales SG&A is set at ~60% of manufacturing costs, following Chandra et al. [71], resulting in a total SG&A allowance of ~90% of manufacturing costs. This allowance likely represents an upper bound for orphan drugs, as small patient populations typically require less promotion and therefore lower sales costs—an observation also noted by Egetis in its 2021 annual report [38].

**Profit:** A profit margin of 8% is applied, aligning with the AIM model (2021) and roughly with the inflation-adjusted average return of the S&P 500 index, adjusted for inflation [40, 72]. Setting the profit margin at this level ensures that returns remain in line with broader market expectations. In the scenarios in Additional file 2, the profit margin is set at 25%, based on a lawsuit filed by the Dutch Pharmaceutical Accountability Foundation against AbbVie over the pricing of Humira®. The case argued that profit margins exceeding 25% of total revenues constitute “excess profits,” potentially diverting resources from other healthcare services [73]. This margin thus presents the threshold above which profit levels may attract legal scrutiny.

**Innovation bonus**: This analysis evaluates the innovation bonus based on AIM criteria (Table 6) and published clinical studies on tiratricol for AHDS (Additional file 4). The value-driven premium is granted only in scenarios assuming no public contribution to tiratricol’s repurposing trajectory (Additional file 2) and is applied to the same cost items used for calculating profit. Given the ongoing uncertainty regarding its precise effectiveness and value, a 30% bonus on total out-of-pocket costs is deemed reasonable.

**Table 6:** Criteria for assessing the effectiveness of a new medical product and assigning an innovation bonus, adapted from AIM (2021) [40]

Criteria:	Innovation bonus (%):
The medicine is indicated for a life-threatening or chronically debilitating or rare disease	5%
The medicine has no alternative	5%
The medicine is curative	30%
Or if the medicine is NOT curative, the following criteria will apply:	
The medicine has shown progression free survival (PFS) gain vs comparator of at least 6 months or at least 50% more than comparator	5%
The medicine has shown overall survival (OS) gain vs comparator of 1 to 6 months	5%
The medicine has shown overall survival (OS) gain vs comparator of more than 6 months	10%
The medicine has shown major quality of life (QOL) improvement	10%

AHDS is a rare and debilitating disorder characterized by severe intellectual and motor disabilities, chronic peripheral thyrotoxicosis, and cerebral hypothyroidism. It qualifies as an indication for a life-threatening, chronically debilitating, or rare disease (5%) [21]. Additionally, no alternative treatments exist; tiratricol is the only effective option for managing AHDS (5%). Other treatments, such as antithyroid drugs, are ineffective or pose significant risks, including hepatotoxicity [20, 22].

Although tiratricol is not curative, it slows disease progression, improving survival and alleviating symptoms such as cardiovascular dysfunction and underweight status, though it does not restore normal neurodevelopment [20, 22]. While no direct comparators exist, tiratricol stabilizes peripheral symptoms, which can be considered comparable to progression-free survival (PFS) gains (5%), as untreated AHDS is progressive and fatal [20].

Regarding overall survival, tiratricol significantly improves outcomes. While exact survival gains in months are not quantified, available evidence suggests a substantial impact, likely exceeding six months (10%) compared to untreated patients [21, 22]. Additionally, tiratricol provides major quality-of-life improvements (10%) by addressing critical symptoms such as chronic thyrotoxicosis, cardiovascular dysfunction, and underweight status, significantly enhancing overall health and well-being [20, 22]. Based on these criteria, the value-driven premium is set at 30% in the maximum scenario, acknowledging pending trial results from the TRIAC II Trial.

**Number of patients:** Egetis projects the total number of patients eligible for treatment to be between 10.000 and 15.000 across the U.S., the EU, and other regions with comparable healthcare standards [38]. This estimate is based on a prevalence of AHDS at 1 in 70.000 males, as reported by Groeneweg et al. (2020) [21]. Using this prevalence and recent figures indicating a total male population of 219 million in the EU [74], this translates to an estimated 3.129 patients who may qualify for treatment.

However, the orphan drug designation granted for tiratricol assumes a significantly smaller patient population of fewer than 500 patients in Europe—the region in this analysis. Therefore, in Scenarios 1a, 1b, 1f, 1 g, and 2a, this lower patient volume is applied. In the other scenarios, the higher estimate is used, as projected by Egetis itself.

### Pricing outcomes

Based on the scenarios depicted in Table 5 and described above, a price PPPY ranging from €5.296 to €27.632 is derived (Table 7). The results for the iterations assuming full private-sector investment and high profit margins are provided in Additional file 2. This operationalisation not only provides a benchmark for healthcare payers to determine a reasonable price for repurposed tiratricol, accounting for the assumptions in each scenario, but also highlights the key components contributing to this price.

**Table 7:** Overview of scenario’s outputs

Items:	Scenario 1a:	Scenario 1b:	Scenario 1c:	Scenario 1d:
**1. Development costs**				
(Net) research & development costs	€ 50.000.000	€ 50.000.000	€ 50.000.000	€ 50.000.000
Cost-of-failure	€ 0	€ 42.000.000	€ 0	€ 42.000.000
**2. Financial correction factors**				
Cost-of-capital	€ 149.824.202	€ 275.676.532	€ 149.824.202	€ 275.676.532
**3. Market-based factor**				
R&D costs, adjusted for regional market share and recoupment period*	€ 1.630.500	€ 1.630.500	€ 1.630.500	€ 1.630.500
Cost-of-failure, adjusted for regional market share and recoupment period*	€ 0	€ 1.369.620	€ 0	€ 1.369.620
Cost-of-capital, adjusted for regional market share and recoupment period*	€ 4.885.767	€ 8.989.812	€ 4.885.767	€ 8.989.812
**4. Annual recurring cost items**				
Manufacturing costs	€ 772.850	€ 772.850	€ 4.836.495	€ 4.836.495
SG&A costs	€ 695.565	€ 695.565	€ 4.352.846	€ 4.352.846
**5. Profit elements**				
Profit	€ 247.913	€ 357.483	€ 865.587	€ 975.157
Innovation bonus	€ 0	€ 0	€ 0	€ 0
**6. Outcomes**				
Price PPPY	€ 16.465	€ 27.632	€ 5.296	€ 7.080

*Reflects the amount that needs to be recouped annually during the recoupment period

**Fig. 2:**
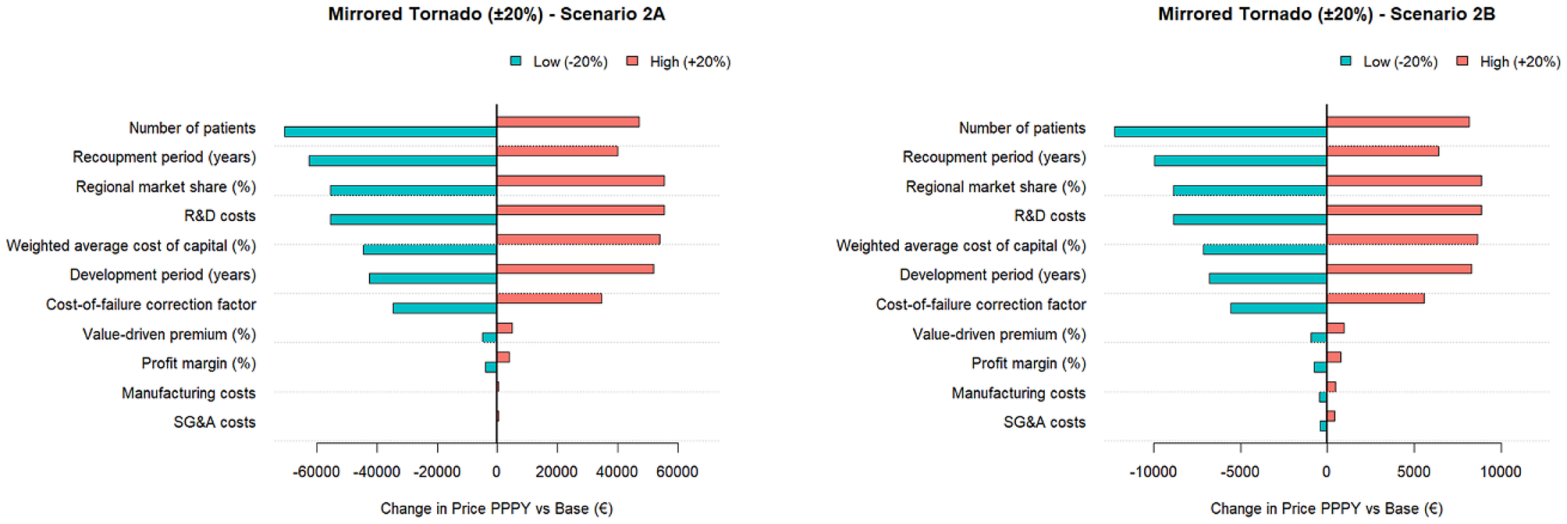
Tornado diagram of sensitivity analysis for scenarios 1a and 1b

**Fig. 3:**
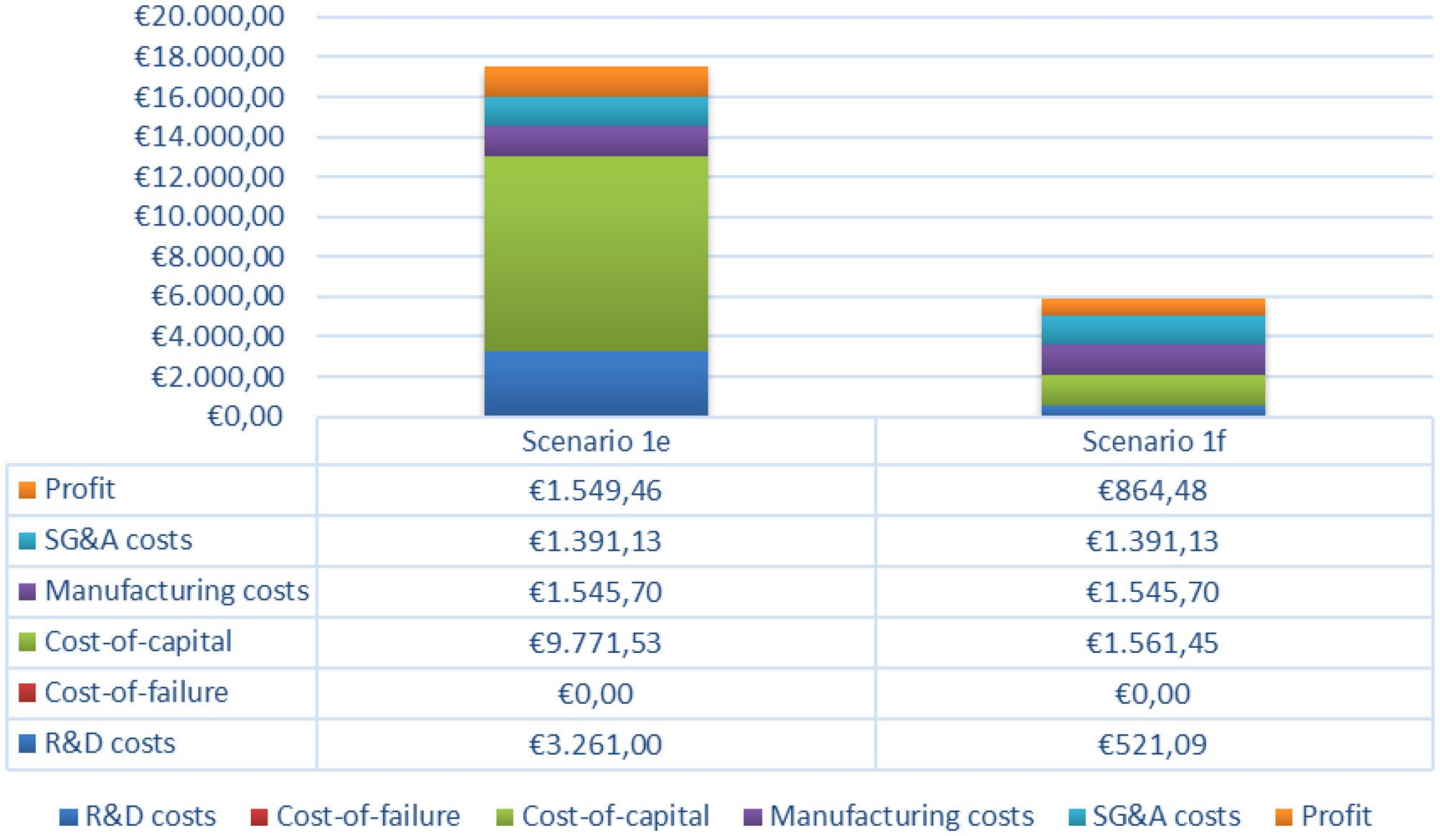
Tornado diagram of sensitivity analysis for scenarios 1c and 1d

**Fig. 4:**
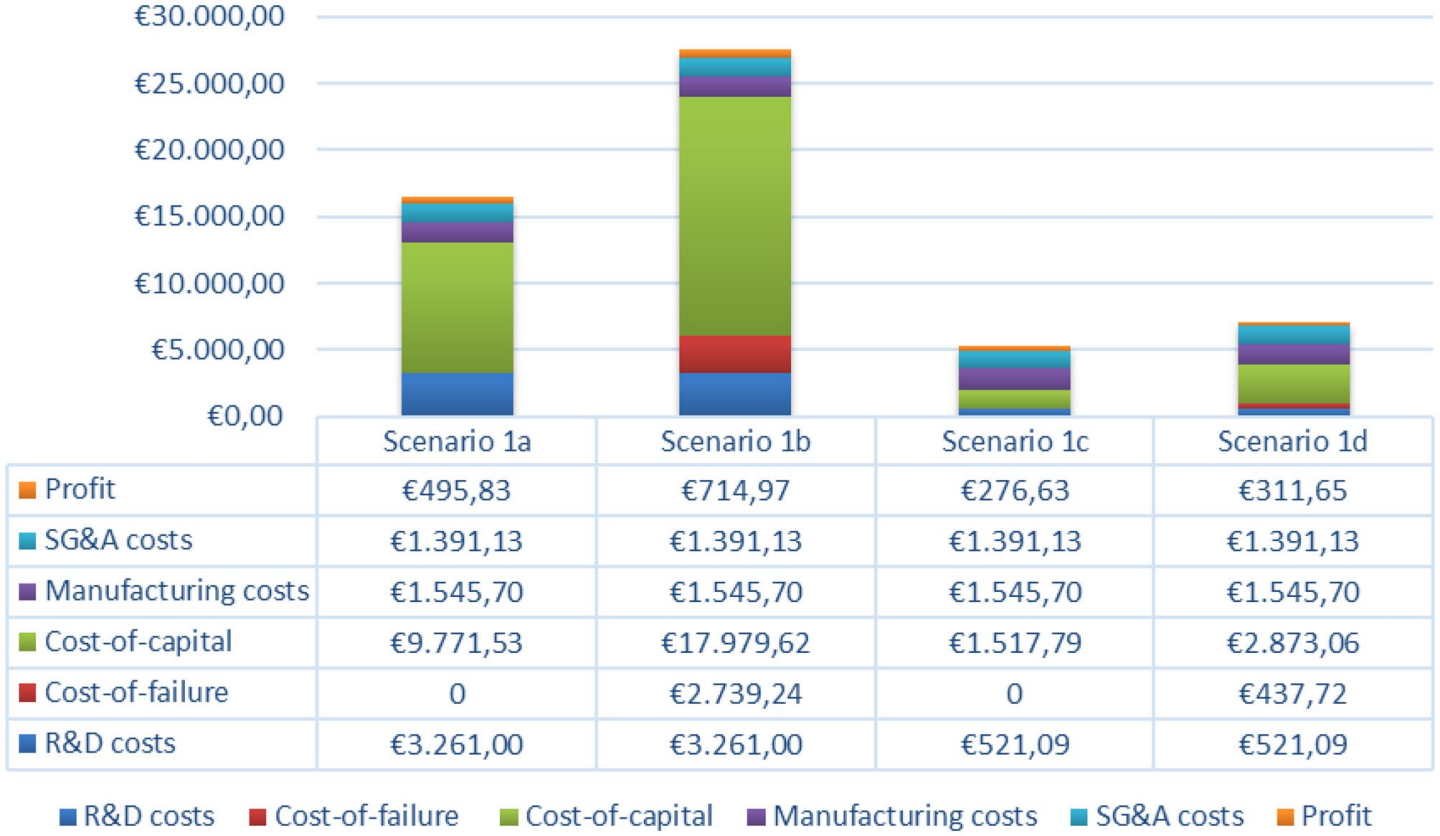
Breakdown of cost components’ relative influence final price PPPY in primary scenarios

Sensitivity analyses conducted RStudio (Additional file 4), as depicted in the tornado charts in the figures below, illustrate both the direction and magnitude of each parameter’s impact on the price PPPY in the highlighted scenarios. The bars represent the effects of a 20% increase and a 20% decrease in each parameter. Figures 2, 3, 4 highlights the relative influence of all cost components on the final price PPPY, breaking down the proportional contribution of each cost component.

The tornado charts show that across all scenarios, the number of patients and the WACC are the most sensitive parameters driving price deviations. Other influential parameters are components related to allocated R&D costs, over which the cost-of-capital is calculated, as well as the development and recoupment periods, which directly affect the cost-of-capital. Together, these findings emphasize the dominant role of fixed development costs and the associated cost-of-capital burden, both of which must be distributed over the number of eligible patients. In contrast, annual recurring cost items such as manufacturing costs, SG&A expenses, and expected profit margins have only a limited effect on price variability.

This pattern aligns with the breakdown of cost components contributing to the final PPPY price. Cost-of-capital exerts by far the largest influence across all scenarios, illustrating the substantial financial impact of capital charges on development investments. These results highlight that pricing outcomes are driven less by operational efficiency and more by financial assumptions regarding return on investments. Interestingly, in scenarios with fewer eligible patients, the primary cost burden arises from upfront development expenditures (R&D costs, cost-of-failure, and cost-of-capital), whereas in scenarios with larger patient populations the cost structure shifts: manufacturing-related expenses become much more prominent in shaping the final PPPY price.

## Discussion

The objective of this analysis is to illustrate how a cost-based-plus pricing framework can offer a transparent structure to guide pricing thresholds and appraisals for repurposed previous low-cost drugs. In this case, the significant role of public and charitable funding in tiratricol’s repurposing, combined with the high expected price set by Egetis, underscores the need to complement health technology assessment with a cost-based pricing approach. A more traditional value-based approach is evident to yield even higher pricing, as demonstrated by the current projected price of Emcitate®. A cost-based-plus price may serve as a benchmark for price negotiations and as basis for a predictable repurposing business case. At the same time, our scenarios show that making orphan drugs available through regulatory approval requires substantial investment, which must be recouped to ensure sustainable accessibility. By adopting a broader perspective on drug pricing, it is possible to balance socially responsible profit margins for manufacturers, acknowledging their investments, while ensuring affordable access to life-saving medicines and safeguarding the sustainability of healthcare budgets.

The primary finding of this study is that when public and charitable contributions are considered, the estimated price for repurposed tiratricol for AHDS is significantly lower than the price projected by Egetis, ranging between €5.296 to €27.632 PPPY, compared to €63.500–€95.000 or higher. Only under the assumption of a fully privately funded repurposing trajectory, combined with a low patient estimate, does the resulting price approach Egetis’s projections (Additional file 2). However, the case analysis indicates that such a privately funded trajectory has not been pursued, given the significant role of public and charitable contributions in tiratricol’s development, most notably through the multinational trial conducted by Erasmus MC [20] and the proof-of-concept studies (Table 3). This raises concerns about whether healthcare systems are effectively paying twice: first through taxpayer-funded research, then through high product prices. More broadly, many therapies rely on decades of public and charitable funding, trial infrastructure, and early-stage research. While these contributions cannot always be fully quantified, failing to account for them risks imposing an additional burden on health systems. The prospect of such a financial burden leads to questions about the appraisal and WTP for repurposed medicines that, as in this case, have been developed with substantial public contributions, as well as the broader implications for access.

If pricing is based solely on therapeutic value, healthcare systems may unintentionally drive costs to unsustainable levels, jeopardizing access to treatment. This is illustrated by Emcitate®’s projected price which exceeds commonly accepted cost-effectiveness thresholds, such as €80,000 per quality-adjusted life year (QALY) in the Netherlands [75]. This outcome is a direct result of applying value-based pricing principles, a strategy the company has explicitly identified as its intended approach in Europe [38]. Such value-based pricing could therefore force healthcare systems to reallocate resources, displacing other essential services or restricting patient access. In practice, payers may seek to bridge this gap through confidential discounts or rebates, which lower the net price but do not resolve the underlying opacity of price setting. Our framework addresses this by offering a transparent benchmark of sustainable pricing, strengthening payers’ negotiating position. Linking prices to actual financial investments provides a counterbalance to purely value-based assessments, safeguarding affordability and access while maintaining incentives for innovation. Rather than replacing traditional HTA, the framework serves as a complementary tool that grounds negotiations in cost transparency while HTA continues to assess value.

The pricing outcomes may entail several limitations arising from the inherent uncertainties associated with estimating a cost-based price without full transparency and from the need to rely on assumptions based on incomplete information. The cost component with the greatest uncertainty is allocated R&D costs. While insights into which aspects of tiratricol’s development for AHDS were co-funded by public institutions allow for more informed estimations, it remains impossible to determine precise R&D costs. Clearly, the R&D allocation applied in this analysis reflects the scope of the current development programme. Should future studies expand beyond the ongoing TRIAC Trial II (NCT02396459) and the ongoing ReTRIACt Study (NCT05579), the model inputs would need to be updated and the resulting prices re-estimated accordingly. However, as long as pharmaceutical companies remain non-transparent about their actual expenditures [76] and estimates for drug development R&D costs vary widely [59–66], the exact costs required to develop an NME or repurpose an existing drug will remain speculative. This highlights the need for greater transparency in drug R&D expenditures, a call already made by various researchers and organizations [77–80]. In this case, over €100 million in development costs may be excessive for a product partly funded by public investment, and even the prices calculated in scenarios 1a-d are significantly lower than the anticipated price for Emcitate®. If the company insists on a higher price, it should provide a transparent and detailed justification of its development costs, including clear documentation of how these costs contribute to the final pricing. While mergers and acquisitions involved in tiratricol’s repurposing may have increased costs that Egetis now seeks to recover, this prompts a broader political and ethical question: To what extent should healthcare payers be responsible for acquisition costs, rebranding, and other business decisions unrelated to clinical development, such as the cost-of-sales, corporate overhead, or returns to investors?

Further questions may arise regarding the patient numbers used in the analyses, particularly because the results indicate that this is a highly sensitive parameter in determining the final price PPPY. However, it is important to note that Egetis itself, based on scientific literature on the prevalence of AHDS [22, 38], projects a higher number of eligible patients than the estimate provided in the orphan drug designation for the EU [27]. Consequently, when applying Egetis’ own estimates, the resulting price is lower due to the greater distribution of fixed and variable costs across a larger patient population. This may indeed become a reality with greater awareness of the disease, driven by treatment availability and the potential inclusion of AHDS in screening programs.

Moreover, while historical manufacturing costs provide a basis for understanding tiratricol’s production costs, it is important to recognize that the production line has likely been modernized, potentially altering these costs. At the same time, it can be argued that the price under DB Pharma’s distribution, used in this analysis, already includes a profit margin for production and distribution. Furthermore, the assumed average patient body weight of 60 kg may be an overestimation, as AHDS is associated with a reduced life expectancy [81]. Consequently, the actual treatment population is likely to consist of younger and lighter patients.

Beyond these considerations, uncertainties remain regarding the values assigned to other cost items, and the extent to which they should be included. The estimated cost-of-failure is based on prior literature [60] but unverifiable without transparency. For tiratricol, development risks were likely limited: a substantial share of the research was publicly funded, much of the risk was absorbed by public institutions, and the compound had already been used in humans for another indication. This suggests the true cost-of-failure may be lower than assumed. It is also debatable whether such costs should be incorporated at all, since doing so could also reward inefficiency. In addition, the SG&A allowance applied here may represent an overestimation, since orphan drugs typically require less promotional effort, an observation also made by Egetis in its 2021 annual report [38]. Conversely, other lifecycle and market-access costs may remain jurisdiction-specific and unobserved within the current SG&A allowance, despite its inclusion to reflect post-authorization commercial activities. Moreover, one may question whether licensing obligations and royalties should be included when assigning profit margins, and whether profits should also be applied to cost-of-failure and cost-of-sales. The weighting of cost items and the assignment of input values is therefore not only an empirical exercise, but also an inherently normative one. These uncertainties illustrate that the assigned costs are open to debate and highlight the need for greater transparency, public discussion, and further analysis to refine cost-based pricing frameworks and increase its validity.

Considering this, the pricing outcomes should not be interpreted as precise figures but rather as reasonable estimates of what the product could and should cost, considering the actual development process. The added value of a cost-based-plus pricing framework, as applied here, does not lie in setting an exact price but in making cost structures transparent, thereby enabling more informed discussions. Such transparency provides industry with a clear upfront expectation of what constitutes a reasonable business case while simultaneously strengthening the negotiating position of healthcare payers. Specifically, for repurposed medicines that were previously inexpensive, this can play a crucial role in price negotiations and in justifying WTP thresholds. By breaking down cost components and providing evidence-based benchmarks, healthcare payers can negotiate more effectively with pharmaceutical companies on how these elements should be weighted and what a product should reasonably cost relative to its development process. In doing so, cost-based pricing can help open the “black box” of drug pricing, shifting the discussion away from whether a single value-based price is “too high” toward examining the underlying structure of a drug’s price—development costs, profit margins, and returns on investment. By explicitly allowing for variable profit margins and optional innovation premiums, the framework can distinguish between cases where significant private investment must be incentivized and those where public contributions have already reduced private risk. This flexibility makes the model directly applicable in payer negotiations, offering a principled basis for differential pricing across products.

Two final remarks must be made regarding the proposed pricing framework. From a healthcare payer’s perspective, it is important to note that the scenarios may assume a recoupment period shorter than the period of market exclusivity. If no competitive products, such as more effective alternatives, enter the market during this time, the initial investment may be recouped before patent expiry and before generic competitors can enter – if they do at all. This suggests an opportunity to establish agreements limiting the duration of higher pricing, aligning pricing strategies more closely with the actual recoupment period to ensure socially responsible and balanced cost recovery. After this period, renegotiations could take place to offer the product at a lower price, closer to actual production costs, in line with proposed two-tier pricing approaches [82, 83]. Additionally, by making pricing components explicit, the framework supports a dynamic and cyclical pricing approach. As more data becomes available on Emcitate®’s effectiveness, production costs, and the eligible patient population, the weight assigned to each pricing component can be adjusted accordingly. This would create a more adaptive pricing structure, ensuring prices reflect the evolving understanding of the drug’s value and cost base over time.

Overall, this study highlights the importance of making clear agreements on pricing and accessibility when universities or public funders collaborate with private companies. In this case, it is evident that failing to establish such agreements can lead to high prices and raise concerns about accessibility. In the case of Egetis, for example, one can rightly question the company’s apparent focus on countries with healthcare standards comparable to those of the Western world, while broader global access to tiratricol should also be a priority. This underscores the need for public entities to secure commitments that prioritize societal value when engaging in partnerships with private firms.

## Electronic supplementary material

Below is the link to the electronic supplementary material.

Supplementary Material 1

## Data Availability

The dataset(s) supporting the conclusions of this article is(are) included within the article (and its Additional file(s)).

## Abbreviations

ADHS: Allan-Herndon-Dudley syndrome
AIM: Alternative Investment Market (London Stock Exchange)
CHMP: Committee for Medicinal Products for Human Use
COGS: Cost-of-goods-sold
EMA: European Medicines Agency
EPAR: European Public Assessment Report
Erasmus MC: Erasmus University Medical Center
EU: European Union
FDA: U.S. Food and Drug Administration
MCT8: Monocarboxylate transporter 8
NME: New molecular entity
OECD: Organisation for Economic Co-operation and Development
PPPY: Per-patient-per-year
PRV: Priority Review Voucher
QALY: Quality-adjusted life year
R&D: Research and development
RPDD: Rare Pediatric Disease Designation
RTH: Resistance to thyroid hormone
RTTI: Rare Thyroid Therapeutics International AB
SG&A: Selling, general and administrative
T3: Triiodothyronine
TRIAC: Triiodothyroacetic acid
WACC: Weighted average cost-of-capital
ZonMW: The Netherlands Organisation for Health Research and Development
