# Supplementary material for: A cost-based-plus pricing approach for repurposed tiratricol in the treatment of Allan-Herndon-Dudley syndrome
Source: Orphanet J Rare Dis. 2026 Mar 5;21:160. doi: 10.1186/s13023-026-04269-7 (PMC13097744; doi:10.1186/s13023-026-04269-7)
Supplement: Supplementary file 1 — Supplementary Material 1 [file 13023_2026_4269_MOESM1_ESM.docx]

# Additional files

## Additional file 1: Allan-Herndon-Dudley Syndrome (AHDS)

AHDS is a rare, X-linked genetic disorder first described by Allan, Herndon, and Dudley in 1944, in a report detailing the sex-linked inheritance of intellectual disability and associated neurological symptoms [1]. The disorder is characterised by severe intellectual disability, developmental delay, and hypotonia in infancy, which progresses to spastic paraplegia and dystonia in later childhood and adulthood [2-5].

AHDS is caused by mutations in the SLC16A2 gene, which encodes Monocarboxylate Transporter 8 (MCT8), a key protein responsible for the cellular uptake of thyroid hormones (THs), including triiodothyronine (T3) and thyroxine (T4) [5]. These mutations impair MCT8 function, disrupting the transport of THs across several tissues, including the blood-brain barrier and neurons, leading to severe hypothyroidism in the brain while peripheral tissues develop hyperthyroidism due to an unsuppressed thyroid axis feedback loop [2]. This dual pathology manifests as profound neurological deficits, including severe intellectual disability and motor dysfunctions, alongside systemic symptoms of thyrotoxicosis, such as tachycardia, hypermetabolism, and muscle wasting [2-5]. THs, particularly the metabolically active T3, are essential for normal neurodevelopment, regulating neurogenesis, neuronal migration and differentiation, synaptogenesis, and myelination [6]. Disruption of these processes in AHDS results in irreversible neurodevelopmental impairments [7]. Another characteristic feature of AHDS is the inability to gain weight despite adequate caloric intake, reflecting the pervasive impact of TH dysregulation on metabolism and muscle integrity [7]. The clinical course varies but typically presents with profound hypotonia in infancy, which may hinder feeding and respiratory function, followed by the development of hypertonia, spasticity, and joint contractures due to chronic motor neuron dysfunction.

Despite these progressive motor symptoms, survival into adulthood is possible, though with significant morbidity. There are currently no curative treatments for AHDS, and management remains supportive, focusing on symptom control and quality of life through a multidisciplinary approach [7-8]. Physical and occupational therapy help manage motor impairments, spasticity, and joint contractures, while speech and feeding therapy address swallowing difficulties and failure to thrive [7]. Due to the risk of aspiration pneumonia and malnutrition, nutritional support, including dietary modifications or gastrostomy tube placement, is often required [8]. Cardiac monitoring is recommended for arrhythmias and tachycardia, and anti-seizure medications may be used for epilepsy, though their effectiveness varies [7].

1. Allan W, Herndon CN, Dudley FC. Some examples of the inheritance of mental deficiency: Apparently sex-linked idiocy and microcephaly. Am J Ment Defic. 1944;48(48):325–34.
2. Schwartz CE, Stevenson RE. The MCT8 thyroid hormone transporter and Allan–Herndon–Dudley syndrome. Best Pract Res Clin Endocrinol Metab. 2007;21(2):307–21.
3. Armour CM, Kersseboom S, Yoon G, Visser TJ. Further insights into the Allan-Herndon-Dudley syndrome: Clinical and functional characterization of a novel MCT8 mutation. PLoS One. 2015;10(10):e0139343.
4. van Geest FS, Groeneweg S, Visser WE. Monocarboxylate transporter 8 deficiency: Update on clinical characteristics and treatment. Endocrine. 2021;71(3):689–95.
5. Groeneweg S, Peeters RP, Moran C, Stoupa A, Auriol F, Tonduti D, et al. Effectiveness and safety of the tri-iodothyronine analogue Triac in children and adults with MCT8 deficiency: An international, single-arm, open-label, phase 2 trial. Lancet Diabetes Endocrinol. 2019;7(9):695–706.
6. Bernal J. Thyroid hormone receptors in brain development and function. Nat Clin Pract Endocrinol Metab. 2007;3(3):249–59.
7. Sarret C, Oliver Petit I, Tonduti D. Allan-Herndon-Dudley syndrome. In: Adam MP, Feldman J, Mirzaa GM, et al., editors. GeneReviews® [Internet]. Seattle (WA): University of Washington, Seattle; 1993–2025. <https://www.ncbi.nlm.nih.gov/books/NBK26373/>. Accessed 3 Feb 2025.
8. Bauer AJ, Auble B, Clark AL, Hu TY, Isaza A, McNerney KP, Sidlow R. Unmet patient needs in monocarboxylate transporter 8 (MCT8) deficiency: A review. Front Pediatr. 2024;12:1444919. <https://doi.org/10.3389/fped.2024.1444919>

## Additional file 2: Secondary analyses

In addition to the four primary analyses that considered the role of public and charitable funding in the repurposing of tiratricol for AHDS, two additional scenarios were conducted assuming a higher profit margin and varying patient numbers (Scenarios 1e–1f). While royalties to former owners are not directly related to development costs, they may influence pricing strategy, since a percentage of net sales must be paid out and companies may therefore apply a higher profit margin. Two further scenarios were analysed under a fully private-investment model, again varying in patient numbers (Scenarios 2a–2b). Table 1 summarizes the assumptions for these scenarios, and Table 2 presents the results with a detailed cost breakdown. Sensitivity analyses were performed for all scenarios (Figures 1 and 2). Figures 3 and 4 illustrate the relative contribution of each cost component to the final PPPY.

Scenarios 1e and 1f, compared with 1a and 1c, show that raising the profit margin has only a modest effect on the final PPPY. This is consistent with the sensitivity analysis and cost breakdown (Figures 1 and 3). In contrast, Scenarios 2a and 2b, which assume a fully private-investment model, demonstrate that R&D costs, cost-of-failure, and cost-of-capital exert a much stronger influence on PPPY (Figure 4). This results from substantially higher assigned values for these components, while annual recurring costs remain constant, increasing the relative weight of fixed development costs. The sensitivity analysis further reveals that the recoupment period is the second most influential parameter. A shorter recoupment period raises the PPPY because investments must be recovered more quickly, but it simultaneously reduces the total capital costs accrued, partially offsetting the increase.

**Table 1:** Overview of scenario’s inputs

| **Items:** | **Scenario 1e:** | **Scenario 1f:** | **Scenario 2a:** | **Scenario 2b:** |
| --- | --- | --- | --- | --- |
| **1. Development costs** | | | | |
| (Net) research & development costs | €50 million | €50 million | €135 million | €135 million |
| Cost-of-failure | None, all risk is presumed to be borne by public entities and charitable foundations | None, all risk is presumed to be borne by public entities and charitable foundations | 1,67 times the assigned (net) R&D costs | 1,67 times the assigned (net) R&D costs |
| **2. Financial correction factors** | | | | |
| Cost-of-capital | Calculated at a discount rate of 10,5% | Calculated at a discount rate of 10,5% | Calculated at a discount rate of 10,5% | Calculated at a discount rate of 10,5% |
| Development period (years) | 10 | 10 | 10 | 10 |
| **3. Market-based factor** | | | | |
| Regional market share (%) | 32,61% | 32,61% | 32,61% | 32,61% |
| Recoupment period (years) | 10 | 10 | 3 | 3 |
| **4. Annual recurring cost items** | | | | |
| Manufacturing costs | Benchmarked at €0,65 per tablet and 2.378 tablets per-patient-per-year | Benchmarked at €0,65 per tablet and 2.378 tablets per-patient-per-year | Benchmarked at €0,65 per tablet and 2.378 tablets per-patient-per-year | Benchmarked at €0,65 per tablet and 2.378 tablets per-patient-per-year |
| SG&A costs | Benchmarked at 90% of manufacturing costs | Benchmarked at 90% of manufacturing costs | Benchmarked at 90% of manufacturing costs | Benchmarked at 90% of manufacturing costs |
| **5. Profit elements** |  | | | |
| Profit (%)* | 25% | 8% | 25% | 25% |
| Innovation bonus* | None, as the value repurposed tiratricol provides to patients primarily stems from public research | None, as the value repurposed tiratricol provides to patients primarily stems from public research | 30% | 30% |
| **6. Patient population** | | | | |
| Number of patients | 500 | 3.129 | 500 | 3.129 |

**Applied to non-capitalized development costs (net R&D costs and cost-of-failure), not adjusted for inflation, but corrected for the regional market share and the assigned recoupment period. This bonus is further granted on top of the out-of-pocket annual recurring cost items. Reflects the annual amount that is provides as bonus each year*

**Table 2:** Overview of scenario’s outputs

| **Items:** | **Scenario 1e:** | **Scenario 1f:** | **Scenario 2a:** | **Scenario 1b:** |
| --- | --- | --- | --- | --- |
| **1. Development costs** | | | | |
| (Net) research & development costs | € 50.000.000 | € 50.000.000 | € 135.000.000 | € 135.000.000 |
| Cost-of-failure | € 0 | € 0 | € 225.450.000 | € 225.450.000 |
| **2. Financial correction factors** | | | | |
| Cost-of-capital | € 149.824.202 | € 149.824.202 | € 720.560.937 | € 720.560.937 |
| **3. Market-based factor** | | | | |
| R&D costs, adjusted for regional market share and recoupment period* | € 1.630.500 | € 1.630.500 | € 14.674.500 | € 14.674.500 |
| Cost-of-failure, adjusted for regional market share and recoupment period* | € 0 | € 0 | € 24.506.415 | € 24.506.415 |
| Cost-of-capital, adjusted for regional market share and recoupment period* | € 4.885.767 | € 4.885.767 | € 78.324.974 | € 78.324.974 |
| **4. Annual recurring cost items** | | | | |
| Manufacturing costs | € 772.850 | € 4.836.495 | € 772.850 | € 4.836.495 |
| SG&A costs | € 695.565 | € 4.352.846 | € 695.565 | € 4.352.846 |
| **5. Profit elements** |  | | | |
| Profit | € 774.729 | € 2.704.960 | € 10.162.333 | € 12.092.564 |
| Innovation bonus | € 0 | € 0 | € 12.194.799 | € 14.511.077 |
| **6. Outcomes** | | | | |
| Price PPPY | € 17.519 | € 5.884 | € 282.663 | € 48.992,93 |

* Reflects the amount that needs to be recouped annually during the recoupment period


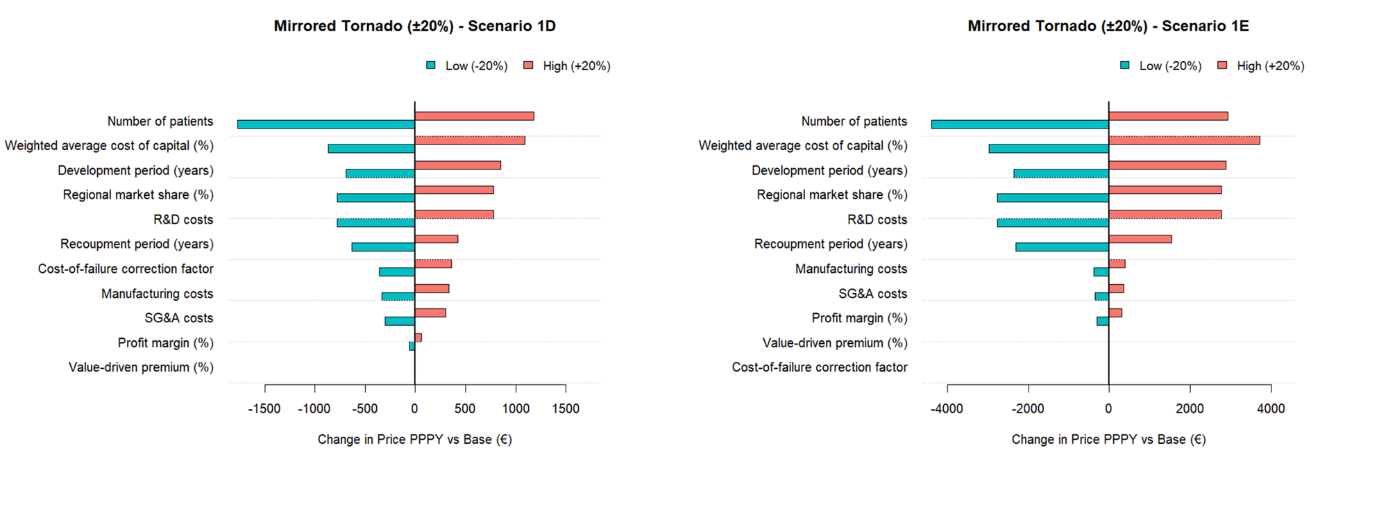


**Figure 1**: Tornado diagram of sensitivity analysis for scenarios 1e and 1f


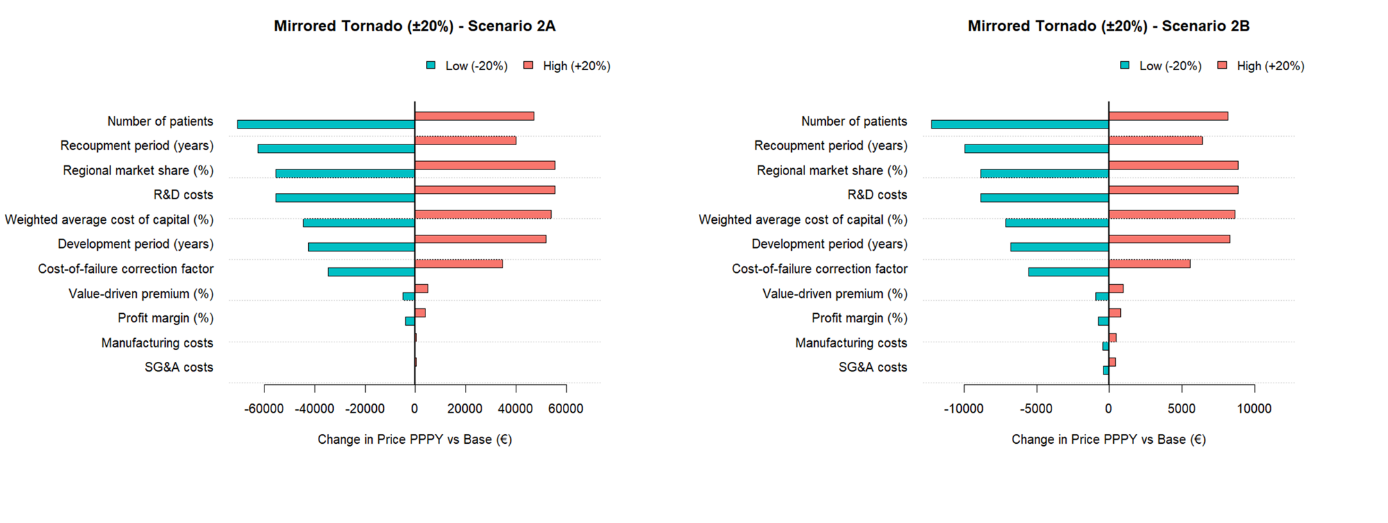

**Figure 2**: Tornado diagram of sensitivity analysis for scenarios 2a and 1b


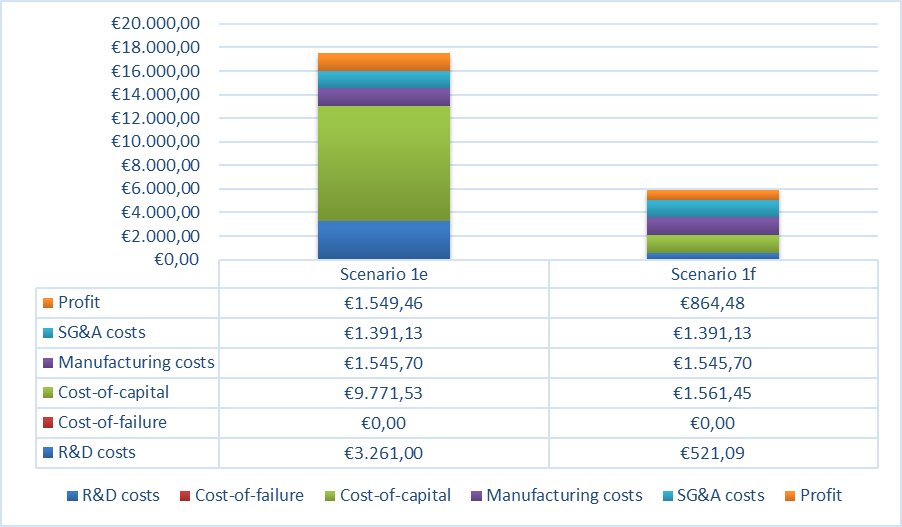


**Figure 3:** Breakdown of cost components’ relative influence final price PPPY in scenario 1e and 1f.

**
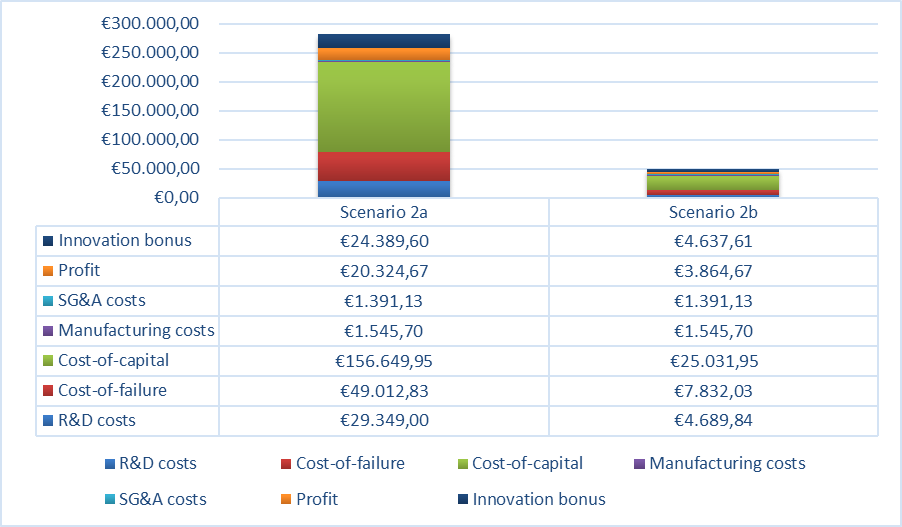
**

**Figure 4:** Breakdown of cost components’ relative influence final price PPPY in scenario 2a and 1b.

## Additional file 3: Overview of registration studies on tiratricol for AHDS

| **Study:** | **Design:** | **Population:** | **Intervention:** | **Primary outcomes:** |
| --- | --- | --- | --- | --- |
| Published and ongoing clinical studies investigating tiratricol for AHDS | | | | |
| Groeneweg et al., 2019 | Phase IIb, open-label, single-arm | 46 male patients with MCT8 deficiency, aged 0.7–66 years | Triac (tiratricol) for 12 months | Assess safety and efficacy in reducing serum T3 levels and improving clinical parameters |
| Groeneweg et al., 2020 | Retrospective, multicenter cohort | 151 males with MCT8 deficiency from 47 centers | Observational study; no intervention | Document natural history and clinical features of MCT8 deficiency |
| van Geest et al., 2022 | Retrospective, real-life cohort | 67 males with MCT8 deficiency, aged 0.6–27.8 years | Triac (tiratricol) with a median treatment duration of 2.7 years | Long-term safety and efficacy in real-life practice |
| TRIAC Trial II (NCT02396459) (ongoing) | Phase II, open-label | 22 male patients with MCT8 deficiency, aged ≤30 months | Triac (tiratricol) for 96 weeks | Assess neurodevelopmental outcomes and safety in young patients |
| ReTRIACt Study (NCT05579327) (ongoing) | Phase III, double-blind, randomized, placebo-controlled | At least 16 male patients, aged ≥4 years | Withdrawal of tiratricol vs. placebo for 30 days | Proportion of patients meeting rescue criteria (serum T3 > upper limit of normal) |
| Studies mentioned in EPAR to support the registration of tiratricol for AHDS | | | | |
| Bioequivalence Study – MCT8-2023-5 | Phase I, randomized, blinded, five-period crossover PK study | 30 healthy adult male volunteers | Single oral doses of tiratricol: 175 µg, 350 µg, 1050 µg (Emcitate-FCP); head-to-head vs Emcitate-DEV at 350 µg; fed/fasted parts | PK and bioequivalence (AUCs, Cmax); food effect; dose proportionality |
| Mouse double knock-out (Mct8/Oatp1c1) study | Non-clinical, in-vivo pharmacology (dKO mouse model) | Mct8/Oatp1c1 double-knockout mice | Tiratricol at doses 50–400 µg/kg/day | Cerebellar development (Purkinje cell dendritogenesis, molecular layer thickness), myelination, gene expression |
| 3-month Juvenile Rat Toxicity Study | Repeat-dose oral toxicity, approximately 13 weeks | Juvenile rats (male and female) | Daily tiratricol at multiple doses (50 µg/kg/day and above not tolerated over 3 months) | Toxicological endpoints (clinical signs, organ weights, histopathology, thyroid hormones) |
| 14-day Dog Toxicity Study | Dose-range finding oral toxicity (2 weeks) | Beagle dogs (male and female) | Tiratricol up to 5 mg/kg/day | Tolerability, clinical chemistry (cholesterol, ALT) |
| 28-day Juvenile Rat Study | Pediatric-specific dose-ranging toxicity (postnatal day 7–34) | Juvenile rats | Tiratricol 0, 50, 150, 450 µg/kg/day (set main-study doses: 0, 50, 100, 300 µg/kg/day) | Growth and development; thyroid hormone suppression; organ weights |
| 13-week GLP Dog Toxicity Study | Repeat-dose oral toxicity (GLP) | Beagle dogs | Tiratricol 0, 0.15, 0.5, 5 mg/kg/day for 13 weeks plus recovery phase | Clinical signs, thyroid hormones, organ weights, histopathology, ECG |

2. Groeneweg S, Peeters RP, Moran C, Stoupa A, Auriol F, Tonduti D, et al. Effectiveness and safety of the tri-iodothyronine analogue Triac in children and adults with MCT8 deficiency: An international, single-arm, open-label, phase 2 trial. Lancet Diabetes Endocrinol. 2019;7(9):695–706. <https://doi.org/10.1016/S2213-8587(19)30155-X>
3. Groeneweg S, van Geest FS, Abacı A, Alcantud A, Ambegaonkar GP, Armour CM, et al. Disease characteristics of MCT8 deficiency: An international, retrospective, multicentre cohort study. Lancet Diabetes Endocrinol. 2020;8(7):594–605. [https://doi.org/10.1016/S2213-8587(20)30153-4](https://doi.org/10.1016/S2213-8587(20)30153-4)
4. van Geest FS, Groeneweg S, van den Akker EL, Bacos I, Barca D, van den Berg SA, et al. Long-term efficacy of T3 analogue Triac in children and adults with MCT8 deficiency: A real-life retrospective cohort study. J Clin Endocrinol Metab. 2022;107(3):e1136–e1147. [https://doi.org/10.1210/clinem/dgab849](https://doi.org/10.1210/clinem/dgab849)
5. ClinicalTrials.gov. Triac Trial II in MCT8 Deficiency Patients (NCT02396459). 2024. [https://clinicaltrials.gov/study/NCT02396459](https://clinicaltrials.gov/study/NCT02396459). Accessed 3 Feb 2025.
6. ClinicalTrials.gov. Withdrawal of Tiratricol Treatment in Males with Monocarboxylate Transporter 8 Deficiency (MCT8 Deficiency) (ReTRIACt) (NCT05579327). 2025. [https://clinicaltrials.gov/study/NCT05579327](https://clinicaltrials.gov/study/NCT05579327). Accessed 3 Feb 2025.
7. European Medicines Agency. Emcitate: EPAR – Public assessment report. 2024. <https://www.ema.europa.eu/en/documents/assessment-report/emcitate-epar-public-assessment-report_en.pdf>. Accessed 3 Feb 2025.

## Additional file 4: R-scripts for sensitivity analysis

############################################################

## Price PPPY (Eq1–Eq11) + ±20% One-Way Sensitivity ##

## Scenarios 1a–1f & 2a–2b | Abs. MC & SG&A | Panels ##

## NEW: Component breakdown plots + CSV per scenario ##

## Outputs: per-scenario CSV/plots + ALL outcomes CSV ##

############################################################

### -------- User options --------

SCENARIO_TO_RUN <- "ALL" # "1a","1b","1c","1d","1e","1f","2a","2b" or "ALL"

SENSITIVITY_PCT <- 0.20 # ±20%

options(stringsAsFactors = FALSE, scipen = 999)

### -------- Helpers --------

`%na0%` <- function(x, y = 0) ifelse(is.na(x), y, x)

as_pct <- function(x) { x <- suppressWarnings(as.numeric(x)); x <- ifelse(is.na(x),0,x); ifelse(x>1,x/100,x) }

ensure_dir <- function(path) { d <- dirname(path); if (!dir.exists(d)) dir.create(d, recursive = TRUE, showWarnings = FALSE) }

euro_fmt <- function(x) formatC(x, format="f", digits=0, big.mark=",")

### -------- Core calculator (returns detailed pieces for overview & breakdown) --------

calc_price_pppy <- function(df_inputs) {

with(df_inputs, {

# CoF from factor (or absolute if supplied)

cof_from_factor <- as.numeric(`Cost-of-failure correction factor` %na0% 0) * as.numeric(`R&Dᵢ` %na0% 0)

`CoFᵢ_eff` <- if ("CoFᵢ" %in% names(df_inputs) && !all(is.na(df_inputs$`CoFᵢ`))) {

as.numeric(df_inputs$`CoFᵢ` %na0% 0)

} else { cof_from_factor }

# Cost of capital (development + recoupment) - inflation handled via GDPᵢ in both phases

wacc <- as_pct(as.numeric(`WACCᵢ` %na0% 0))

gdp <- as_pct(as.numeric(`GDPᵢ` %na0% 0))

D <- as.numeric(`Dᵢ` %na0% 0)

R <- as.numeric(`Rᵢ` %na0% 0)

base_RD_CoF <- as.numeric(`R&Dᵢ` %na0% 0) + `CoFᵢ_eff`

CoC_Development_vec <- base_RD_CoF * ((1 + wacc)^D * (1 + gdp)^D - 1)

CoC_Recoup_vec <- vapply(seq_along(R), function(i) {

if (R[i] <= 0) return(0)

sum((base_RD_CoF[i] + CoC_Development_vec[i]) *

(1 - (1:R[i]) / R[i]) * wacc[i] * (1 + gdp[i])^(1:R[i]))

}, numeric(1))

CoC_vec <- CoC_Development_vec + CoC_Recoup_vec

# Market allocation & annual cost items

MAF_vec <- as_pct(as.numeric(`Regional market share` %na0% 0)) / pmax(R, 1)

if (!"Manufacturing costs" %in% names(df_inputs)) stop("Need 'Manufacturing costs' column (absolute €).")

MC_vec <- as.numeric(df_inputs$`Manufacturing costs` %na0% 0)

SGA_vec <- as.numeric(df_inputs$`SG&Aᵢ` %na0% 0)

# Basis for profit & innovation (Eq10-11)

basis_vec <- ((as.numeric(`R&Dᵢ`) + `CoFᵢ_eff`) * MAF_vec) + MC_vec + SGA_vec

profit_m <- as_pct(as.numeric(`Profit margin` %na0% 0))

IB_m <- as_pct(as.numeric(`Value-driven premium` %na0% 0))

P_vec <- profit_m * basis_vec

IB_vec <- IB_m * basis_vec

# Numerator for Eq1 and PPPY

numer_vec <- ((as.numeric(`R&Dᵢ`) + `CoFᵢ_eff` + CoC_vec) * MAF_vec) +

MC_vec + SGA_vec + P_vec + IB_vec

denom <- pmax(as.numeric(`Patientsᵢ` %na0% 0), 1)

PPPY <- sum(numer_vec) / sum(denom)

list(

Price_PPPY = PPPY,

# pieces used later

RnD = sum(as.numeric(`R&Dᵢ` %na0% 0)),

CoF = sum(`CoFᵢ_eff`),

RnD_CoF = sum(as.numeric(`R&Dᵢ` %na0% 0) + `CoFᵢ_eff`),

CoC_dev = sum(CoC_Development_vec),

CoC_rec = sum(CoC_Recoup_vec),

CoC_total = sum(CoC_vec),

MAF = mean(MAF_vec),

RnD_MAF = sum(as.numeric(`R&Dᵢ` %na0% 0) * MAF_vec),

CoF_MAF = sum(`CoFᵢ_eff` * MAF_vec),

CoC_MAF = sum(CoC_vec * MAF_vec),

MC = sum(MC_vec),

SGA = sum(SGA_vec),

Basis_sum = sum(basis_vec),

Profit_amt = sum(P_vec),

Innov_amt = sum(IB_vec),

CoF_sum = sum(`CoFᵢ_eff`),

CoC_sum = sum(CoC_vec)

)

})

}

### -------- One-way sensitivity --------

one_way_sa <- function(df_inputs, vary_fields, pct = 0.20) {

base <- calc_price_pppy(df_inputs)$Price_PPPY

res <- lapply(vary_fields, function(fld) {

low_df <- df_inputs; high_df <- df_inputs

scale_field <- function(x, mult) {

if (is.numeric(x)) return(x * mult)

nx <- suppressWarnings(as.numeric(x))

if (all(!is.na(nx))) return(nx * mult)

x

}

low_df[[fld]] <- scale_field(low_df[[fld]], 1 - pct)

high_df[[fld]] <- scale_field(high_df[[fld]], 1 + pct)

low <- calc_price_pppy(low_df)$Price_PPPY

high <- calc_price_pppy(high_df)$Price_PPPY

data.frame(Component=fld, Base=base, Low=low, High=high, Low_Δ=low-base, High_Δ=high-base, check.names=FALSE)

})

do.call(rbind, res)

}

### -------- Labels for tornado plots --------

label_map <- c(

"R&Dᵢ" = "R&D costs",

"Cost-of-failure correction factor" = "Cost-of-failure correction factor",

"WACCᵢ" = "Weighted average cost of capital (%)",

"Dᵢ" = "Development period (years)",

"Regional market share" = "Regional market share (%)",

"Rᵢ" = "Recoupment period (years)",

"Manufacturing costs" = "Manufacturing costs",

"SG&Aᵢ" = "SG&A costs",

"Profit margin" = "Profit margin (%)",

"Value-driven premium" = "Value-driven premium (%)",

"Patientsᵢ" = "Number of patients"

)

apply_labels <- function(sa) {

sa$Display <- label_map[sa$Component]

sa$Display[is.na(sa$Display)] <- sa$Component

sa

}

### -------- Tornado plotters (most sensitive at TOP + clean legend) --------

save_abs_tornado <- function(sa, pct, scenario_tag, out_dir = ".") {

sa <- apply_labels(sa)

effect <- pmax(abs(sa$Low_Δ), abs(sa$High_Δ))

# Most sensitive at TOP: sort increasing (barplot draws first element at bottom)

ord <- order(effect, decreasing = FALSE)

sa <- sa[ord, ]

effect <- effect[ord]

png_file <- file.path(out_dir, paste0("scenario_", scenario_tag, "_tornado_abs.png"))

ensure_dir(png_file)

png(png_file, width=1200, height=800, res=120)

op <- par(mar=c(7,22,6,4))

barplot(effect, horiz=TRUE, names.arg=sa$Display, las=1,

xlab="Absolute change in Price PPPY (€)",

main=paste0("Tornado (absolute, ±", round(100*pct), "%) - Scenario ", toupper(scenario_tag)))

grid(nx=NA, ny=NULL)

par(op); dev.off()

png_file

}

save_mirrored_tornado <- function(sa, pct, scenario_tag, out_dir = ".") {

sa <- apply_labels(sa)

effect <- pmax(abs(sa$Low_Δ), abs(sa$High_Δ))

# Most sensitive at TOP: sort increasing

ord <- order(effect, decreasing = FALSE)

sa <- sa[ord, ]

left_vals <- -abs(sa$Low_Δ)

right_vals <- abs(sa$High_Δ)

vals <- rbind(left_vals, right_vals)

x_max <- max(abs(vals), na.rm=TRUE) * 1.05

png_file <- file.path(out_dir, paste0("scenario_", scenario_tag, "_tornado_mirrored.png"))

ensure_dir(png_file)

png(png_file, width=1400, height=900, res=120)

# CHANGED: more top margin so legend can sit above plot without overlapping bars

op <- par(mar=c(9,28,10,6))

bp <- barplot(vals, horiz=TRUE, beside=TRUE, col=c("#00BFC4","#F8766D"),

xlim=c(-x_max, x_max), names.arg=rep("", length(left_vals)*2), las=1,

xlab="Change in Price PPPY vs Base (€)",

main=paste0("Mirrored Tornado (±", round(100*pct), "%) - Scenario ", toupper(scenario_tag)))

grid(nx=NA, ny=NULL)

abline(v=0, lwd=2)

y_centers <- colMeans(bp)

axis(2, at=y_centers, labels=sa$Display, las=1, tick=FALSE)

# CHANGED: legend placed in top margin (clean, no overlap with bars)

usr <- par("usr")

par(xpd=NA)

legend(x = mean(usr[1:2]), y = usr[4] + 0.18 * (usr[4] - usr[3]),

legend = c(paste0("Low (-", round(100*pct), "%)"), paste0("High (+", round(100*pct), "%)")),

fill = c("#00BFC4", "#F8766D"),

horiz = TRUE, bty = "n", cex = 0.95)

par(xpd=FALSE)

par(op); dev.off()

png_file

}

save_pair_panel <- function(sa_left, tag_left, sa_right, tag_right, pct, filename) {

sa_left <- apply_labels(sa_left)

sa_right <- apply_labels(sa_right)

# Most sensitive at TOP: sort increasing

ord_l <- order(pmax(abs(sa_left$Low_Δ), abs(sa_left$High_Δ)), decreasing = FALSE)

ord_r <- order(pmax(abs(sa_right$Low_Δ), abs(sa_right$High_Δ)), decreasing = FALSE)

sa_left <- sa_left[ord_l, ]

sa_right <- sa_right[ord_r, ]

png(filename, width=1900, height=900, res=120)

# CHANGED: extra top margin for a shared legend above both panels

par(mfrow=c(1,2), mar=c(9,28,10,6), oma=c(0,0,3,0))

vals_l <- rbind(-abs(sa_left$Low_Δ), abs(sa_left$High_Δ))

x_max_l <- max(abs(vals_l), na.rm=TRUE) * 1.05

bp <- barplot(vals_l, horiz=TRUE, beside=TRUE, col=c("#00BFC4","#F8766D"),

xlim=c(-x_max_l, x_max_l), names.arg=rep("", ncol(vals_l)*2), las=1,

xlab="Δ Price PPPY (€)", main=paste0("Scenario ", toupper(tag_left)))

grid(nx=NA, ny=NULL); abline(v=0, lwd=2)

axis(2, at=colMeans(bp), labels=sa_left$Display, las=1, tick=FALSE)

vals_r <- rbind(-abs(sa_right$Low_Δ), abs(sa_right$High_Δ))

x_max_r <- max(abs(vals_r), na.rm=TRUE) * 1.05

bp <- barplot(vals_r, horiz=TRUE, beside=TRUE, col=c("#00BFC4","#F8766D"),

xlim=c(-x_max_r, x_max_r), names.arg=rep("", ncol(vals_r)*2), las=1,

xlab="Δ Price PPPY (€)", main=paste0("Scenario ", toupper(tag_right)))

grid(nx=NA, ny=NULL); abline(v=0, lwd=2)

axis(2, at=colMeans(bp), labels=sa_right$Display, las=1, tick=FALSE)

# Title + shared legend in outer margin (clean, no overlap)

mtext(paste0("Mirrored Tornado (±", round(100*pct), "%)"), outer=TRUE, line=1, cex=1.2)

par(xpd=NA)

legend("top", inset=c(0, -0.02),

legend = c(paste0("Low (-", round(100*pct), "%)"), paste0("High (+", round(100*pct), "%)")),

fill = c("#00BFC4", "#F8766D"),

horiz = TRUE, bty = "n", cex = 0.95)

par(xpd=FALSE)

par(mfrow=c(1,1)); dev.off()

}

### -------- NEW: Component breakdown table + plot --------

build_breakdown <- function(df) {

c <- calc_price_pppy(df)

data.frame(

Item = c(

"R&D costs",

"Cost-of-failure",

"R&D costs plus cost-of-failure",

"Cost-of-capital (development phase), adjusted for inflation",

"Cost-of-capital (recoupment phase), adjusted for inflation",

"Cost-of-capital (development & recoupment phase), adjusted for inflation",

"R&D costs, adjusted for market size and recoupment period*",

"Cost-of-failure, adjusted for market size and recoupment period*",

"Cost-of-capital (development & recoupment phase), adjusted for market size, recoupment period, and inflation*",

"Manufacturing costs",

"Sales, general, and administrative (SG&A) costs",

"Profit",

"Innovation bonus",

"Price PPPY"

),

EUR = c(

c$RnD,

c$CoF,

c$RnD_CoF,

c$CoC_dev,

c$CoC_rec,

c$CoC_total,

c$RnD_MAF,

c$CoF_MAF,

c$CoC_MAF,

c$MC,

c$SGA,

c$Profit_amt,

c$Innov_amt,

c$Price_PPPY

),

check.names = FALSE

)

}

save_breakdown_plot <- function(df, scenario_tag, out_dir = ".") {

tb <- build_breakdown(df)

png_file <- file.path(out_dir, paste0("scenario_", scenario_tag, "_component_breakdown.png"))

ensure_dir(png_file)

ord <- seq_len(nrow(tb))

vals <- tb$EUR[ord]

names(vals) <- tb$Item[ord]

xmax <- max(vals[1:13], na.rm = TRUE) * 1.15

png(png_file, width=1200, height=900, res=120)

op <- par(mar=c(8, 32, 6, 4))

bp <- barplot(vals, horiz=TRUE, las=1,

names.arg = names(vals),

xlim = c(0, xmax),

xlab = "Euros (€)",

main = paste0("Component Breakdown - Scenario ", toupper(scenario_tag)))

grid(nx=NA, ny=NULL)

text(x = vals + xmax*0.01, y = bp, labels = euro_fmt(vals), adj = 0, cex = 0.8)

mtext("* Adjusted by regional market share and recoupment period (MAF).", side=1, line=5, cex=0.9)

par(op); dev.off()

utils::write.csv(tb, file = paste0("scenario_", scenario_tag, "_component_breakdown.csv"), row.names = FALSE)

png_file

}

### -------- Scenario builders --------

build_scn <- function(patients, r_and_d_m, cof_factor, recoup_yrs, profit_m, value_prem,

wacc=10.5, dev_yrs=10, mshare=32.61, gdp=0) {

MC <- 0.65 * 2378 * patients

SGA <- 0.90 * MC

data.frame(

check.names = FALSE,

`R&Dᵢ` = r_and_d_m * 1e6,

`Cost-of-failure correction factor` = cof_factor,

`WACCᵢ` = wacc,

`Dᵢ` = dev_yrs,

`Rᵢ` = recoup_yrs,

`GDPᵢ` = gdp,

`Regional market share` = mshare,

`Manufacturing costs` = MC,

`SG&Aᵢ` = SGA,

`Profit margin` = profit_m,

`Value-driven premium` = value_prem,

`Patientsᵢ` = patients

)

}

# 1a–1d (8% profit, 0% premium; CoF none or 0.84×; R=10)

scn_1a <- build_scn( 500, 50, NA, 10, 0.08, 0.00)

scn_1b <- build_scn( 500, 50, 0.84, 10, 0.08, 0.00)

scn_1c <- build_scn(3129, 50, NA, 10, 0.08, 0.00)

scn_1d <- build_scn(3129, 50, 0.84, 10, 0.08, 0.00)

# 1e–1f (25% profit, 0% premium; CoF none; R=10)

scn_1e <- build_scn( 500, 50, NA, 10, 0.25, 0.00)

scn_1f <- build_scn(3129, 50, NA, 10, 0.25, 0.00)

# 2a–2b (25% profit, 30% premium; CoF 1.67×; R=3)

scn_2a <- build_scn( 500, 135, 1.67, 3, 0.25, 0.30)

scn_2b <- build_scn(3129, 135, 1.67, 3, 0.25, 0.30)

scenario_map <- list(

`1a`=scn_1a, `1b`=scn_1b, `1c`=scn_1c, `1d`=scn_1d,

`1e`=scn_1e, `1f`=scn_1f, `2a`=scn_2a, `2b`=scn_2b

)

scenarios_to_run <- if (toupper(SCENARIO_TO_RUN)=="ALL") names(scenario_map) else SCENARIO_TO_RUN

if (any(!scenarios_to_run %in% names(scenario_map))) stop("SCENARIO_TO_RUN must be any of 1a–1f,2a–2b or 'ALL'.")

### -------- Sensitivity fields --------

vary_these <- c("R&Dᵢ","Cost-of-failure correction factor","WACCᵢ","Dᵢ",

"Regional market share","Rᵢ","Manufacturing costs","SG&Aᵢ",

"Profit margin","Value-driven premium","Patientsᵢ")

### -------- Run scenarios, save plots/CSVs, build overview --------

sa_store <- list()

overview <- data.frame(

Scenario=character(), Patients=integer(), RnD_EUR=numeric(), CoF_factor=numeric(),

WACC=numeric(), DevYrs=integer(), RecoupYrs=integer(), MarketShare=numeric(),

MC_EUR=numeric(), SGA_EUR=numeric(), ProfitMargin=numeric(), ValuePremium=numeric(),

CoF_EUR=numeric(), CoC_EUR=numeric(), Basis_EUR=numeric(),

Profit_EUR=numeric(), Innovation_EUR=numeric(), Price_PPPY=numeric()

)

for (tag in scenarios_to_run) {

df <- scenario_map[[tag]]

# Baseline components

calc <- calc_price_pppy(df)

# Sensitivity & plots

sa <- one_way_sa(df, vary_these, SENSITIVITY_PCT)

sa_store[[tag]] <- sa

utils::write.csv(sa, file=paste0("scenario_", tag, "_sensitivity.csv"), row.names=FALSE)

save_abs_tornado(sa, SENSITIVITY_PCT, tag)

save_mirrored_tornado(sa, SENSITIVITY_PCT, tag)

# Component breakdown (plot + CSV)

save_breakdown_plot(df, tag)

# Append to overview

overview <- rbind(overview, data.frame(

Scenario = tag,

Patients = df$`Patientsᵢ`[1],

RnD_EUR = df$`R&Dᵢ`[1],

CoF_factor = df$`Cost-of-failure correction factor`[1],

WACC = df$`WACCᵢ`[1],

DevYrs = df$`Dᵢ`[1],

RecoupYrs = df$`Rᵢ`[1],

MarketShare = df$`Regional market share`[1],

MC_EUR = df$`Manufacturing costs`[1],

SGA_EUR = df$`SG&Aᵢ`[1],

ProfitMargin = df$`Profit margin`[1],

ValuePremium = df$`Value-driven premium`[1],

CoF_EUR = calc$CoF_sum,

CoC_EUR = calc$CoC_sum,

Basis_EUR = calc$Basis_sum,

Profit_EUR = calc$Profit_amt,

Innovation_EUR= calc$Innov_amt,

Price_PPPY = calc$Price_PPPY

))

cat("Scenario ", toupper(tag), " - Price PPPY: ",

formatC(calc$Price_PPPY, format="f", digits=2, big.mark=","), " €\n", sep="")

}

### -------- Side-by-side tornado panels --------

make_if_available <- function(a,b,filename){

if (all(c(a,b) %in% names(sa_store))) {

save_pair_panel(sa_store[[a]], a, sa_store[[b]], b, SENSITIVITY_PCT, filename)

}

}

make_if_available("1a","1b","panel_1a_1b_tornado.png")

make_if_available("1c","1d","panel_1c_1d_tornado.png")

make_if_available("1e","1f","panel_1e_1f_tornado.png")

make_if_available("2a","2b","panel_2a_2b_tornado.png")

### -------- Save combined overview --------

utils::write.csv(overview, file="outcomes_overview_all.csv", row.names=FALSE)

cat("\n================= ALL OUTCOMES =================\n"); print(overview)

cat("Saved combined overview: ", normalizePath("outcomes_overview_all.csv", winslash="/"), "\n")

cat("Saved per-scenario component plots/CSVs: scenario_*_component_breakdown.png/.csv\n")

cat("Saved tornado panels (if scenarios present): panel_1a_1b_tornado.png, panel_1c_1d_tornado.png, panel_1e_1f_tornado.png, panel_2a_2b_tornado.png\n")

cat("\nAll done. 🍪\n")
